# Comparative *In silico* Analysis of Butyrate Production Pathways in Gut Commensals and Pathogens

**DOI:** 10.3389/fmicb.2016.01945

**Published:** 2016-12-02

**Authors:** Swadha Anand, Harrisham Kaur, Sharmila S. Mande

**Affiliations:** Bio-Sciences R&D Division, TCS Research, Tata Consultancy Services Ltd.Pune, India

**Keywords:** butyrate production pathways, gut microbiome, butyrate producers, genome mining, comparative genomics

## Abstract

Biosynthesis of butyrate by commensal bacteria plays a crucial role in maintenance of human gut health while dysbiosis in gut microbiome has been linked to several enteric disorders. Contrastingly, butyrate shows cytotoxic effects in patients with oral diseases like periodontal infections and oral cancer. In addition to these host associations, few syntrophic bacteria couple butyrate degradation with sulfate reduction and methane production. Thus, it becomes imperative to understand the distribution of butyrate metabolism pathways and delineate differences in substrate utilization between pathogens and commensals. The bacteria utilize four pathways for butyrate production with different initial substrates (Pyruvate, 4-aminobutyrate, Glutarate and Lysine) which follow a polyphyletic distribution. A comprehensive mining of complete/draft bacterial genomes indicated conserved juxtaposed genomic arrangement in all these pathways. This gene context information was utilized for an accurate annotation of butyrate production pathways in bacterial genomes. Interestingly, our analysis showed that inspite of a beneficial impact of butyrate in gut, not only commensals, but a few gut pathogens also possess butyrogenic pathways. The results further illustrated that all the gut commensal bacteria (*Faecalibacterium, Roseburia, Butyrivibrio*, and commensal species of *Clostridia* etc) ferment pyruvate for butyrate production. On the contrary, the butyrogenic gut pathogen *Fusobacterium* utilizes different amino acid metabolism pathways like those for Glutamate (4-aminobutyrate and Glutarate) and Lysine for butyrogenesis which leads to a concomitant release of harmful by-products like ammonia in the process. The findings in this study indicate that commensals and pathogens in gut have divergently evolved to produce butyrate using distinct pathways. No such evolutionary selection was observed in oral pathogens (*Porphyromonas* and *Filifactor*) which showed presence of pyruvate as well as amino acid fermenting pathways which might be because the final product butyrate is itself known to be cytotoxic in oral diseases. This differential utilization of butyrogenic pathways in gut pathogens and commensals has an enormous ecological impact taking into consideration the immense influence of butyrate on different disorders in humans. The results of this study can potentially guide bioengineering experiments to design therapeutics/probiotics by manipulation of butyrate biosynthesis gene clusters in bacteria.

## Introduction

Humans harbor a plethora of micro-organisms comprising of around 1000 species inhabiting different body sites. Some of them are beneficial bacteria which help not only in metabolism and absorption of nutrients by the human host but also in regulation of our immune system (Bhattacharya et al., 2015). These bacteria can also influence epithelial cell growth and differentiation (Schwabe and Jobin, 2013; Sears and Garrett, 2014). The human body sites with the most diverse microbiome are gut followed by oral cavity (Corpet et al., 1995). Recent studies have indicated that microbiome is influenced by various environmental factors. Any insult to the critical balance of the microbiome composition, resulting in the outgrowth of harmful bacteria, might be responsible for triggering diseases/disorders like inflammatory bowel disease (IBD), diabetes, obesity, periodontitis etc. (Gupta et al., 2011; Ghosh et al., 2014; Jorth et al., 2014; Tomasello et al., 2014). Recent studies have also indicated that the onset of disease cannot be accredited to a single pathogen, but to the entire microbiome (Schwabe and Jobin, 2013).

The human gut maintains the most diverse microbiome comprising of bacteria which are beneficial for health and have an anti-inflammatory effect on intestinal epithelium (Basson et al., 2000). Majority of these bacteria produce metabolites like short chain fatty acids (SCFA) that are known to be beneficial for the host. For example, previous studies have indicated increased butyric acid in the stool samples of healthy individuals as compared to those suffering from enteric diseases (Basson et al., 2000). Similarly, while no significant differences were seen in propionate level, an elevated acetate level was observed in the gut of disease cohorts (Weir et al., 2013). These results suggest that amongst the three bacteria-derived SCFA’s, butyrate might play a significant role in determining the gut health status of an individual. This is supported by higher occurrence of butyrate producing bacteria, like *Faecalibacterium, Coprococcus*, and *Roseburia* in the guts of healthy individuals (Hakansson and Molin, 2011; Sun and Chang, 2014). On the contrary, the abundances of these genera were observed to be lower in the guts of individuals with CRC, IBD, ulcerative colitis, diabetes, etc. (Dulal and Keku, 2014). Reports have also indicated that administering butyrate can affect the production of cyclin D3 (Siavoshian et al., 2000; Tang et al., 2011), which may lead to a cessation of cell in G1 phase of cell cycle and a shift toward terminal differentiation. Butyrate is experimentally shown to be a histone deacetylase inhibitor, further emphasizing its role in reducing cell proliferation by epigenetic regulation (Bordonaro et al., 2014; Donohoe et al., 2014). Production of butyrate has been shown to decrease the pH and has been proposed to prevent the growth of pathogenic organisms like *Enterococcus* and *Escherichia* in the gut (Duncan et al., 2009; Slavin, 2013). These studies suggest that butyrate produced by gut bacteria has a positive influence on gut health. Studies have indicated that butyrate obtained from natural dietary fiber can help maintain gut homeostasis and reduce the idiopathies of various diseases that develop due to dysbiosis (Toden et al., 2014).

Another human body site known to be colonized by a wide diversity of bacteria is the oral cavity. Earlier studies have shown that contrary to its role in gut, butyrate has a cytotoxic effect on gingival cells of humans and proves to be pathogenic in oral environment (Ohkawara et al., 2005). Butyrate has also been shown to be responsible for release of Reactive Oxygen species in chronic periodontitis (Chang et al., 2013). Further, it has been implicated in apoptosis and autophagic cell death in gingival cells (Ohkawara et al., 2005). The dysbiosis within oral microbiome is often associated with an increase in butyrate producing pathogens like *Porphyromonas gingivalis, Filifactor alocis*, and *Tannerella forsythia* and has also been implicated in diseased conditions like periodontitis (Corpet et al., 1995; Socransky et al., 1998; Aruni et al., 2015). Thus, while butyrate is a beneficial metabolite for gut cells, its presence is likely to show deleterious effects in oral cavity. These differences in roles of butyrate in different body sites in humans necessitate a deeper understanding of butyrate production in various bacteria. In addition, delineating differences in butyrate production mechanisms of commensals and pathogens is likely to help in designing better probiotics for improving gut/oral health.

**FIGURE 1:**
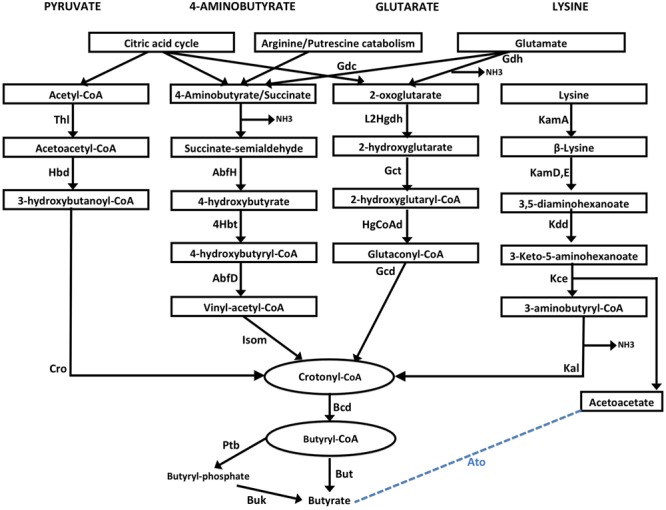
**Schematic representation of four butyrate production pathways in bacteria.** Pyruvate pathway: Pyruvate is converted to crotonyl CoA using three enzymes, namely, Thiolase (Thl), Hydroxybutyryl dehydrogenase (Hbd) and crotonase/enoyl-CoA hydratase (Cro). 4-aminobutyrate (4Ab) pathway: 4Ab is converted to crotonyl CoA by the action of AbfH (4-hydroxybutyrate dehydrogenase), 4Hbt (butyryl-CoA:4-hydroxybutyrate-CoA transferase) and AbfD (4-hydroxybutyryl dehydratase) which also possesses vinyl-acetyl-CoA isomerase activity. Glutarate pathway: 2-oxoglutarate conversion to Crotonyl-CoA involves 2-hydroxyglutarate dehydrogenase (L2Hgdh), glutaconate-CoA transferase (Gct) and 2-hydroxyglutaryl-CoA dehydrogenase (HgCoAd) and Glutaconyl-CoA decarboxylase (Gcd). Glutamate can be converted to 4-aminobutyrate and 2-oxoglutarate by enzymes Glutamate decarboxylase (Gdc) and Glutamate dehydrogenase (Gdh) enzymes. Lysine pathway: Lysine is metabolized to Crotonyl-CoA by lysine 2,3-aminomutase (KamA), lysine 5,6-aminomutase (Kam D,E), 3,5-diaminohexanoate dehydrogenase (Kdd), 3-keto-5-aminohexanoate cleavage enzymes (Kce) and 3-aminobutyryl-CoA ammonia lyase (Kal). Acetoacetate released in the last step can also be converted to Butyate by a few bacteria using butyryl-CoA:acetoacetate-CoA transferase (Ato) enzyme. Crotonyl-CoA, a product from each of the four pathways, is metabolized to butyryl-CoA by butyryl-CoA dehydrogenase (Bcd). Conversion of butyryl-CoA to butyrate is catalyzed by either two enzymesphosphate butyryl transferase (Ptb) and Butyrate kinase (Buk), or by butyryl-CoA:acetate CoA transferase (But).

Four major butyrate production pathways exist in bacteria (**Figure 1**). These pathways utilize one of the four substrates namely, pyruvate, glutarate, 4-aminobutyrate and lysine. Each of these four pathways use butyryl-CoA dehydrogenase electron-transferring flavoprotein complex (Bcd-Etfαβ) to catalyze conversion of crotonyl-CoA to butyryl CoA (Chowdhury et al., 2014). Eventually the final production of butyrate is catalyzed by either butyryl-CoA:acetate CoA transferase (But) or butyrate kinase (Buk) (**Figure 1**). It should also be noted that Glutamate is used by anaerobic bacteria for production of the substrates 4-aminobutyrate and 2-oxoglutarate while these substrates can be produced as intermediates of citric acid cycle in aerobic bacteria. Further, arginine catabolism can also be used in certain bacteria to biosynthesize 4-aminobutyrate.

One of the *in silico* studies has utilized just the presence of But and Buk enzymes (terminal enzymes) as markers for predicting butyrate production capability by bacteria (Vital et al., 2013). Similarly, homology-based analysis has been utilized in another study to identify existence of all genes involved in the butyrate production in various bacterial genomes (Vital et al., 2014). It is to be noted that both these methods utilize sequence homology for prediction of genes. Since some of the genes involved in the four butyrate pathways are known to be utilized in other metabolic pathways, the homology-based analyses is expected to lead to misannotations. In order to overcome this drawback, apart from identifying homologs of these genes, it is important to consider their genomic locations in the organisms. The clustered genomic arrangement of genes comprising each of these butyrate producing pathways has also been reported earlier in a few bacteria (Boynton et al., 1996; Li et al., 2012; Whon et al., 2015).

In the present study, a comprehensive Hidden Markov Model (HMM) based genomic analysis was performed to understand distribution of butyrate production pathways in commensals and pathogens inhabiting different environments. Firstly, the genomic arrangement of these pathways was confirmed and this context information was utilized for identifying domains within the cognate butyrate production pathway in 8027 bacterial genomes. The utilization of juxtaposed genome organization of domains comprising a pathway enabled elimination of homologous domains which might have different functions in other metabolic pathways also. This study, to the best knowledge, is the first to shed light on the distribution of butyrate production pathways in pathogens versus commensals across different environmental conditions like gut and oral cavity in humans. Further, an attempt was made to understand similarities/differences in butyrate production capabilities as well as pathways utilized for biosynthesis by gut pathogens and commensals. In order to further verify our findings, publicly available 16S rRNA amplicon datasets, corresponding to gut samples from 443 healthy and 567 diseased individuals, were analyzed. The results, based on the gene context information (from bacterial genomes) in combination with the taxonomic composition, provided valuable insights into the differences in evolution of butyrate production pathways in commensals and pathogens in gut. The genome-wide study further gave insights into the distribution of butyrate production pathways in bacteria inhabiting the oral cavity in periodontitis patients as the butyrogenic pathways differed in strains forming the oral microbiome of these individuals.

## Results

### Distribution of Butyrate Production Pathways in Bacteria

**FIGURE 2:**
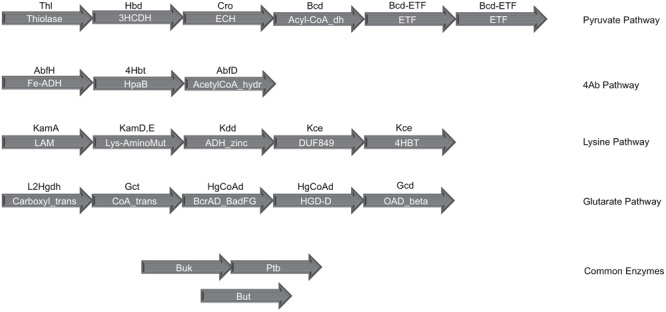
**Gene organization of the four butyrate production pathways in bacteria.** The clustered genomic organization of four butyrate production pathways has been depicted. The text within the arrows shows the PFAM domain assignments corresponding to each gene in a pathway while the text above the arrow indicates the gene identifier for which PFAMs were used. Butyryl-CoA dehydrogenase (Bcd), the central enzyme in all four pathways, occurs in genomic context with the Pyruvate pathway genes and contains the dehydrogenase as well as Electron transferring α and β subunits. The final enzymes phosphate butyryl transferase (Ptb), butyrate kinase (Buk) and butyryl-CoA:acetate CoA transferase (But) occur on different locations in the bacterial genomes.

In order to evaluate butyrate production capability in various bacteria, the four known pathways that are utilized by them for butyrate production (**Figure 1**) were studied. A preliminary analysis using HMM based approach (details in Section “Materials and Methods”) was performed on known butyrate producers (e.g., *Faecalibacterium, Roseburia, Fusobacteria, Coprococcus*) to confirm the evolutionary conservation of clustered gene arrangement for all four butyrogenic pathways. The identified juxtaposed arrangement of genes (**Figure 2**) constituting butyrate production pathways is in agreement with previously reported studies (Boynton et al., 1996; Li et al., 2012; Whon et al., 2015). Although, the domain order for each pathway differed across genomes, all genes of a pathway were found to occur in context with each other. After establishing the conserved genomic arrangement of butyrate production pathways, this HMM based analysis was extended to 8027 sequenced genomes (complete and draft) in NCBI to understand butyrate pathway composition across all bacteria. Results of the analyses indicated presence of one or more of these pathways in butyrate producing organisms. 2180 out of 8027 bacteria were found to have all genes that are involved in butyrate production from pyruvate. The remaining three pathways utilizing 4-Aminobutyrate (4Ab), Lysine and Glutarate, were observed to be present in 41, 110 and 46 genomes, respectively. Thus, the majority of butyrate producers were found to utilize pyruvate as substrate.

### Role of Electron Transfer Proteins in Butyryl-CoA Dehydrogenase of Butyrogenic Bacteria

The Bcd (butyryl-CoA dehydrogenase) enzyme is known to be a common component utilized by all butyrate producers (**Figure 1**). Interestingly, while this gene was found to occur in context with genes constituting the pyruvate pathway, it was found to function in *trans* in other three pathways. The Bcd protein is known to be composed of three subunits, namely, α-subunit which acts as butyryl-CoA dehydrogenase (Bcd) and the β- and γ-subunits which function as electron-transferring flavoproteins (ETF) (Chowdhury et al., 2014). While all subunits of this protein were observed to be present in anaerobic butyrate producers, the β- and γ-subunits were found to be absent in aerobic butyrate producing bacteria (Supplementary Tables S1 and S2). Interestingly, majority of the bacteria which showed presence of ETF proteins (β- and γ-subunits) in addition to dehydrogenase component (α-subunit) were observed to be mostly gut commensals including *Faecalibacterium, Roseburia, Eubacterium, Odoribacter, Oscillibacter*, and *Butyrivibrio*. The ETF + dehydrogenase complex was also observed in oral pathogens like *Porphyromonas gingivalis* and *Filifactor alocis*. These pathogens lead to butyrate production in oral cavity which, as discussed earlier, is cytotoxic in this environment and may lead to disorders like periodontitis (Socransky et al., 1998; Tsuda et al., 2010; Chang et al., 2013; Aruni et al., 2015). Apart from these bacteria residing in human microbiome, a few strains of *Azotobacter vinelandii*, a bacteria known to thrive in soil environment and those of the thermophillic *Thermoanaerobacteria* were observed to possess all three subunits of Bcd, which is in line with earlier studies (Ueno et al., 2006). Many sulfate reducing bacteria, identified to possess pyruvate pathway like *Desulfitobacterium*, have been reported to utilize butyrate as an electron donor for sulfate reduction (Gerritse et al., 1999). Further, studies have hypothesized the role of ETFs (in Bcd enzymes) in energy conservation in anaerobic bacteria (Herrmann et al., 2008). Thus, the results from our analysis indicate that butyrate biosynthesis might be coupled with energy conservation in anaerobic butyrate producers.

Since some of the enzymes involved in the above mentioned four butyrate producing pathways are present in multiple copies on the genome, the gene context-based information utilized in the present study (**Figure 2**) ensured accurate annotation of all butyrogenic pathways. The predicted butyrate producers (Supplementary Table S1) from completely sequenced genomes indicated that not all strains of bacteria were capable of butyrate biosynthesis. For example, only five out of 18 strains of *Lachnospiracea* were found to have one or more of the butyrate pathways. Results also indicated that different strains of a bacterial species produce butyrate utilizing different pathways. For example, while *Fusobacterium nucleatum vincentii* was found to possess the 4-aminobutyrate pathway, the other strains of *Fusobacteria* contained Glutarate and Lysine pathways. Thus, the findings of the present study are expected to help in identifying strains of bacteria that are capable of producing butyrate.

### Butyrate Producers in Human Gut

In order to evaluate the role of butyrogenic bacteria in human gut, publicly available datasets for 16S rRNA amplicons corresponding to 443 healthy and 567 diseased individuals were considered. Results of the multivariate data analysis using the bootstrapped abundance values (details in Section “Materials and Methods”), indicated higher presence of *Firmicutes* and *Bacteroidetes* in all populations, irrespective of the health status of the individuals. The butyrate producing *Firmicutes* were found to have higher abundances in healthy subjects across all demographies. For example, genus *Faecalibacterium* was seen to have three to fourfold higher abundances in all healthy subjects, which is in line with earlier reported experimental studies (Castellarin et al., 2012; Kostic et al., 2012; Warren et al., 2013; El-Semman et al., 2014). Other genera which were observed to be high in some of the healthy samples included *Dorea, Blautia, Flavonifractor, Roseburia, Clostridia, Gemmiger, Coprococcus, Erysipelotrichacea, and Butyricimonas* (Supplementary Figure S1). Eight of the above mentioned 11 genera, prevalent in healthy individuals, are known to produce butyrate that is useful for gut health. Thus, the higher abundance of butyrate producers in healthy individuals suggests the beneficial effect of butyrate on gut health and well being in humans. It is to be noted that the healthy datasets showed a higher abundance of *Lachnospiracea_incertae_sedis*. The term ‘incertae sedis’ refers to strains of family *Lachnospiracea* which cannot be assigned to any genus based on the 16S rRNA sequence homology. Many of these strains have been reported to be potential butyrate producers which contribute to healthy gut microbiota (Clarke et al., 2013; Jiang et al., 2015).

The results of the multivariate analyses obtained using 567 diseased patients showed higher abundance of genera like *Streptococcus, Anaerococcus, Veillonella, Escherichia, Rothia, Campylobacter, Leptotrichia*, and *Fusobacterium* (Supplementary Figure S1). Interestingly, *Fusobacterium* is the only butyrogenic genus which showed twofold higher occurrence in all the patients suffering from CRC (95 samples) and IBD (101 samples). Also, although butyrogenic genus *Megasphaera* was not observed in the guts of the subjects considered in this study, it has been reported to have higher abundance in many diseased conditions (Bajaj et al., 2012; Ling et al., 2013; Chiu et al., 2014). The presence of butyrogenic bacteria in the guts of diseased individuals calls for an in-depth comparison of various butyrate production pathways in likely pathogens and commensals.

### Gut Bacteria and Butyrate Production Pathways

#### Comparison of Butyrate Production Pathways in Commensals and Pathogens

**FIGURE 3:**
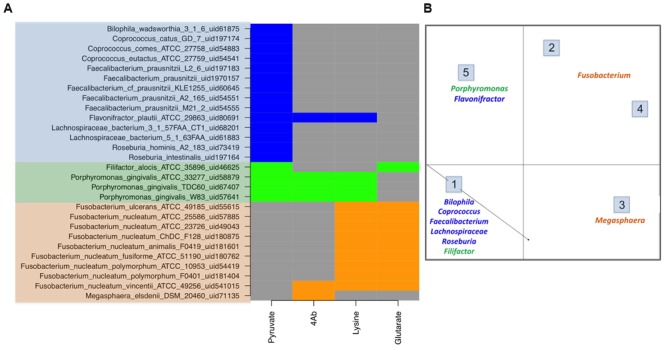
**Butyrate production pathway composition in commensals vs. pathogens. (A)** The figure depicts the pathway distribution in bacteria which form a part of human microbiome. The gut commensals (blue), gut pathogens (orange) and oral pathogens (green) show differential presence of butryogenic pathways. **(B)** Clustering of human microbiome bacteria on the basis of pathway presence.

The genome mining for four different butyrate production pathways was performed on all sequenced bacterial genomes (as described earlier). On mapping the genera showing differential abundance in healthy versus diseased datasets to the catalog of butyrate production pathways within each genome (Supplementary Table S1), different distributions were observed (**Figure 3A**).

The results of the analyses indicated the presence of pyruvate pathway genes involved in butyrate production in genera which are majorly observed in healthy cohorts, namely *Faecalibacterium, Flavonifractor, Roseburia, Coprococcus*, and *Butyricimonas*. Another group of bacteria, showing higher abundance in these cohorts, corresponded to *Lachnospiracea incertae sedis.* Thus, we attempted to find butyrate production pathway distribution within the *Lachnospiracea* strains which are known to inhabit gut environs. The analysis indicated that most sequenced gut *Lachnospiracea* strains lack butyrate production pathways, indicating incapability to produce beneficial butyrate. These strains included *Lachnospiracea 8_1_57FAA_uid61885, Lachnospiracea 1_1_57FAA_uid68209, Lachnospiracea 6_1_63FAA_uid66423, Lachnospiracea 2_1_46FAA_uid66429, Lachnospiracea 9_1_43 BFAA_uid66425, Lachnospiracea 4_1_37FAA_uid63581, Lachnospiracea 1_4_56FAA_uid68205, Lachnospiracea 2_1_58FAA_uid68203* and *Lachnospiracea 5_1_57FAA_uid68199*. The only strains of *Lachnospiraceae* which inhabit the gut and carry pyruvate pathway genes for butyrate production are *Lachnospiraceae_bacterium_5_1_63FAA_uid61883* and *Lachnospiraceae_bacterium_3_1_57FAA_CT1*. Interestingly, while only these two strains show phylogenetic similarity to known butyrogenic gut bacteria (*Roseburia intestinalis* and *Coprococcus comes*), all other strains of *Lachnospiraceae* found in gut belong to a different clade. This was also in agreement with previous study indicating lack of butyrate pathways in many strains of *Lachnospiracea* inhabiting the gut (Meehan and Beiko, 2014). The present study also indicates that all strains of *Roseburia* (*Roseburia intestinalis* and *Roseburia hominis*), *Faecalibacterium* (*Faecalibacterium prausnitzii*), *Butyrivibrio* (*Butyrivibrio fibrisolvens*), *Odoribacter* (*Odoribacter splanchnicus*), which were observed as commensals in healthy gut by earlier studies (Ohkawara et al., 2005; Duncan et al., 2006; Morgan et al., 2012; Heinken et al., 2014), are capable of producing butyrate using pyruvate pathway. Thus, this study provides an insight into strains within various genera which may be responsible for butyrate production in gut. It should be noted here that this analysis helps to delineate distribution of butyrate production pathways in strains which belong to highly abundant genera in human gut. It does not imply that all these strains would be present in a collected sample, but indicates which strains within a genus, if present, can possibly influence the gut health (in terms of butyrate production and the pathways utilized to biosynthesize the same). It is important to note that majority of the publicly available metagenomic data for comparison of taxonomic changes in healthy and diseased cohorts is based on 16S rRNA which allows classification of gut microbiota composition mostly at genus level. Thus, the exact constituent strains of gut microbiota cannot be established through 16S rRNA metagenomic analysis.

Pyruvate is known to be biosynthesized by the glucose-metabolizing glycolytic pathway in all bacteria. However, the results of the present analyses suggest that only butyrogenic commensals flux some of this pyruvate to bring about butyrate production. Also, it was observed that gut commensals like *Flavonifractor* and few strains of *Lachnospiracea* possessed not only pyruvate pathway, but also other butyrate biosynthetic pathways (**Figure 3A**). In addition, **Figure 3A** shows that known gut commensals like *Odoribacter* and *Oscillibacter* (although not observed in any of the datasets under study) also show the presence of pyruvate pathway.

Contrary to the commensal bacteria, butyrate biosynthesis pathways were not identified in majority of pathogenic genera that were highly abundant in the gut of diseased cohorts. These genera (described earlier) include all strains of *Enterococcus, Streptococcus, Escherichia, Campylobacter* and *Leptotrichia*. On the other hand, the three remaining pathways (4Ab, Glutarate and Lysine) were found in only 0.2% of differentially abundant genera present in healthy subjects. Interestingly, while the 4Ab pathway was seen in *Fusobacteria* and *Megasphaera*, glutarate and lysine pathways were identified only in *Fusobacteria*. Although a few strains of *Fusobacterium nucleatum* have been reported as oral pathogens, a number of invasive strains have been associated with gastrointestinal diseases also (Allen-Vercoe et al., 2011). All these gut associated strains of *Fusobacterium* **Figure 3A**) lack Pyruvate pathway for butyrate production. In addition studies have indicated *Fusobacterium varium* to be present in the gut of diseased individuals (Ohkusa et al., 2002). Based on the present analyses, lysine and glutarate pathways were found only in one strain of *Fusobacteria*, namely *Fusobacterium_nucleatum_ATCC_25586_uid57885* (**Figure 3A**). Similarly, *Megasphaera elsdenii*, another butyrogenic organism, was found to utilize only 4Ab pathway. The abundance of this genus has been reported to increase in case of diseased conditions like obesity, hepatic encephalopathy and bacterial vaginosis (Bajaj et al., 2012; Ling et al., 2013; Chiu et al., 2014). Thus, the results of the present study indicate possible correlation between butyrogenic gut bacteria that belong to genera which show higher abundance in diseased condition and lack of the pyruvate pathway. It is also important to note that although pyruvate utilizing bacterial genera like *Faecalibacterium, Roseburia* and *Lachnospiracea incertae sedis* (explained above) were present in diseased datasets; their abundances were less than half as compared to those in healthy datasets. Another observation from this analysis emphasizes that although 4Ab, Lysine and Glutarate pathway might be present in commensal bacteria, they were found to always occur in addition to Pyruvate pathway. Therefore, specific presence of pyruvate pathway in commensals emphasizes its importance in differentiating commensals from pathogens. **Figure 3B** shows clustering for human microbiome associated butyrogenic bacteria on the basis of pathway distribution. The results showed that commensal bacteria formed a separate cluster distinct from pathogenic ones. Cluster 1 constituted gut commensals (for which genome sequences are available) which possessed 100% pyruvate pathway. Interestingly, the oral pathogens *Filifactor* and *Porphyromonas* also clustered with these gut commensals. On the other hand, Cluster 2 comprised of *Megasphaera* and was observed to have 100% of its strains utilizing 4Ab pathway for butyrogenesis. While Lysine and Glutarate pathways for butyrate production were observed to occur in 100% of pathogenic *Fusobacterium* strains in Clusters 3 and 4, only 11% of *Fusobacterium* strains of Clusters 3 and 4 were found to possess 4Ab pathway. Cluster 5 contained 100% of sequenced gut commensal *Flavonifractor* and oral pathogen *Porphyromonas* strains possessing 4Ab, Lysine and Pyruvate pathways. The presence of oral pathogens in both Clusters 1 and 5 indicated no specific preference for pathway utilization in these genera. It should be pointed out here that these percentages are limited to the sequenced strains of these bacteria in NCBI.

Pathogenic species of *Clostridia* were found to be the exception to the above mentioned observation regarding lack of pyruvate pathway for butyrate production in pathogenic genera. These species were found to possess all four genes of this pathway (Supplementary Table S1). Further analysis showed that these bacterial species are capable of converting butyrate to butanol, a pathway absent in all gut commensals. Interestingly, butanol is known to regulate endospore formation and production of toxins in pathogenic *Clostridia* (Karlsson et al., 2000, 2008; Ling et al., 2014).

#### Butyrate Production and Ammonia Release in Diseased Gut

*Fusobacteria*, one of the genera observed in the guts of individuals with certain diseases/disorders, were found to contain butyrate production pathways. However, results of the present study indicated that instead of pyruvate, *Fusobacterium* utilizes 4Ab, glutarate and lysine pathways for butyrate production (Supplementary Figure S2). A detailed genomic analysis of pathways indicated absence of Bcd enzyme (critical enzyme) (**Figure 1**) in majority of *Fusobacteria* strains. The only exception was found to be *Fusobacterium_nucleatum_ATCC_25586*, a strain known to be a common pathogen in periodontal infections (Han et al., 2000). Interestingly, this strain was found to have the Bcd gene flanked by transposase, suggesting possible acquisition of this gene through horizontal gene transfer event. However, this gene was not found to be located in the vicinity of the genes belonging to butyrate pathways. Since Bcd is a common domain which can be utilized in different pathways, the presence of this domain in only a single strain of *Fusobacterium* thus suggests its involvement in pathways other than butyrate.

Out of the three butyrate production pathways in *Fusobacteria*, 4Ab and Glutarate pathways are known to utilize 4-aminobutyrate (succinate being the precursor) and 2-oxoglutarate as starting substrates respectively. Since anaerobic bacteria like *Fusobacteria* lack citrate acid cycle which produces these substrates as intermediates, they are likely to utilize Glutamate to form 2-oxoglutarate and 4-aminobutyrate. Thus, the production of 2-oxoglutarate can only is brought about by dehydrogenation of Glutamate by Glutamate dehydrogenase in *Fusobacteria*, with release of ammonia in the process. Earlier studies have shown that metabolism of amino acids in the gut might lead to an increase in pathogenic bacteria like *Escherichia, Enterococcus* etc. (Richardson et al., 2013). Also, higher ammonia in the gut has been shown to increase chances of CRC (Corpet et al., 1995; Xu et al., 2015). Hence, the ammonia released as a by-product of butyrate pathways in pathogens probably impacts gut health. It is to be noted that although, these studies show the detrimental effect of ammonia released during protein fermentation, correlation of gut health with ammonia released during butyrate production by pathogens has not been reported earlier. The different consequences of butyrate production by commensals and by pathogens on gut health have also not been elucidated in literature.

The third butyrate production pathway observed in *Fusobacterium* utilizes lysine as a substrate. The enzymes for lysine biosynthesis are known to be absent in this genus. Interestingly, results of the genome-context analyses indicated presence of a lysine permease in the vicinity of the gene cluster coding for lysine pathway for butyrate production in *Fusobacterium nucleatum ATCC 25586*. This suggests that lysine utilized in this pathway for butyrate production by this strain is probably obtained from the host. This in turn leads to a competition with host for lysine, an essential amino acid for the humans. In addition to extracting an essential resource from the host, this pathway also leads to release of ammonia which is deleterious to the gut health.

Apart from the above mentioned three pathways, previous studies have also reported involvement of methylaspartate pathway in butyrate production in *Fusobacterium varium* (Ramezani et al., 2011), a pathogen observed in human ulcerative colitis. This pathway (Supplementary Figure S2), involved in fermentation of Glutamate to Acetyl-CoA and finally Butyryl CoA, has been observed in various strains of *Fusobacteria* (Ramezani et al., 2011). Further analysis in this study on sequenced genomes (data not shown) showed that amongst the butyrate producers, this pathway was observed in *Fusobacteria* and few pathogenic strains of Clostridium like *Clostridium tetani* and *Clostridium tetanophorum*. The occurrence of this pathway was completely absent in other butyrogenic gut commensals. This pathway also produces ammonia while converting Glutamate to Acetyl CoA. Combining the above observations, the present study illustrates that the butyrogenic pathways which utilize amino acids as initial substrates (glutarate, 4Ab, Lysine, methyl aspartate) are prevalent in gut pathogens and lead to ammonia production that is harmful for gut health.

**FIGURE 4:**
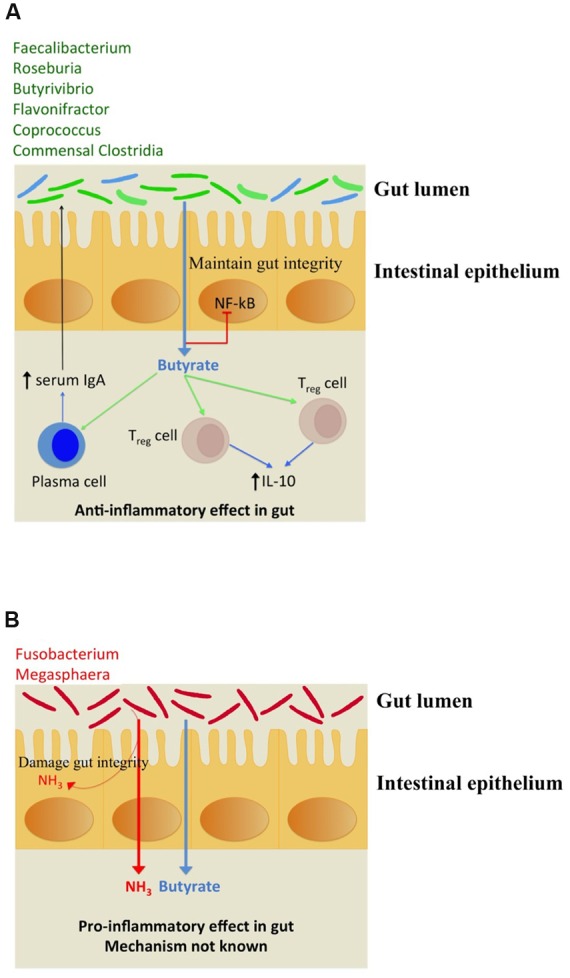
**(A)** Depiction of beneficial anti-inflammatory immune response triggered by butyrate biosynthesis in commensal bacteria (shown in green). The commensal bacteria not capable of producing butyrate have been depicted in blue. **(B)** Pro-inflammatory immune response to ammonia released along with butyrogenesis by gut pathogens.

The immunomodulation effects of butyrate toward anti inflammatory response have been studied. It stimulates regulatory T cells (Treg) to increase the production of IL-10 which is an anti-inflammatory cytokine as depicted in **Figure 4A**. Butyrate also stimulates plasma cells to secrete serum IgA which limits the proliferation of pathogenic bacteria in gut lumen. The pathogenic bacteria which possess butyrogenic pathways lead to a concomitant ammonia production which increases inflammation in gut as explained above (**Figure 4B**). Thus, this study indicates that pathogens retain butyrogenic pathways which bring about release of harmful ammonia which might cause damage to gut integrity leading to higher probability of pathogen invasion.

### Butyrate Producers and Pathways in Oral Cavity

As discussed earlier, another observation from the genome wide study of butyrate production pathways was their presence in pathogens implicated in oral diseases like periodontitis. The role of butyrate in oral mucosa is contrasting to that in gut as it is known to be cytotoxic to these cells (Tsuda et al., 2010). It has also been proven that butyrate in oral cells might lead to production of Reactive Oxygen Species which might lead to inflammation (Chang et al., 2013). These butyrogenic oral pathogens include *Fusobacterium nucleatum* (discussed earlier in this manuscript) which primarily utilizes amino acid metabolism for butyrate production (Jorth et al., 2014). Additionally, other oral pathogenic bacteria like *Porphyromonas gingivalis, Tannerella forsythia* and *Filifactor alocis* have also been shown to possess butyrate production pathways (Socransky et al., 1998; Darveau, 2010; Aruni et al., 2015). Our study revealed that while all strains of *Porphyromonas gingivalis* possessed the Pyruvate, 4-aminobutyrate and Lysine pathways for butyrate production, *Filifactor alocis* contained Pyruvate and Glutarate pathways and *Tannerella forsythia* possessed the 4-Aminobutyrate pathway for butyrate production. This finding is in agreement with an earlier metatranscriptomic study which showed an upregulation of pyruvate fermentation, lysine catabolism and glutamate catabolism in chronic periodontis patients as compared to healthy cohorts (Jorth et al., 2014). Thus, these results show that contrary to observations for gut pathogens, oral pathogens do not show any pathway level preference and possess pyruvate as well as aminoacid fermenting pathways.

The above observations indicate that an environment like oral cavity, where Butyrate itself is cytotoxic, does not present a selection of a particular set of pathways for pathogenic bacteria. Even the pyruvate fermentation pathway, which does not release any ammonia, can be utilized by these pathogens to release butyrate which can itself have a deletrious effect in diseases like chronic periodontitis.

## Discussion

**FIGURE 5:**
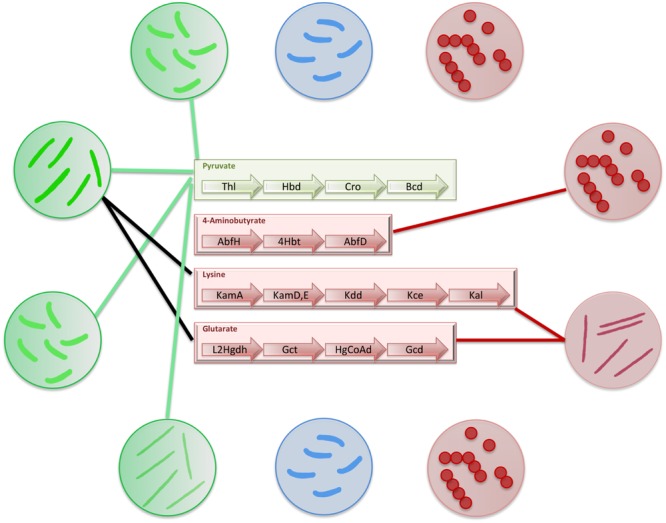
**Distribution of butyrate production pathways in gut bacteria.** The figure depicts the distribution of butyrate production pathways in gut bacteria. The commensal bacteria which produce butyrate have been depicted in green circles and connections have been shown to mark the butyrate production pathways present in each of them. The commensal bacteria which do not produce butyrate have been depicted in blue circles. The pathogenic bacteria have been depicted in red circles. The connections to corresponding butyrogenic pathways have been made to depict pathogens that produce butyrate.

The present study indicates not only increased abundance of butyrogenic commensal bacteria in healthy cohorts, but also reveals that majority of them utilize pyruvate as initial substrate for butyrate production. On the contrary, most of the pathogenic bacteria do not possess butyrate production pathways. Interestingly, *Fusobacteria* which was noticed to be highly abundant in diseased gut cohorts (CRC and IBD patients), was the only pathogenic genus observed in gut to possess butyrate producing capability. Several strains of *Fusobacterium* (known to be oral or gut pathogens) utilize 4Ab, Glutarate and Lysine pathways for butyrate production (**Figure 5**). It is interesting to note that this pathogen lacks Citrate or Glyoxylate pathways whose intermediates (succinate and 2-oxoglutarate, respectively) act as substrates for the first two pathways (4Ab and Glutarate). Similarly, this pathogen also lacks lysine biosynthesis genes for producing substrate for the third pathway (Lysine). Thus, this pathogenic bacteria probably utilizes either its amino acid metabolic pathways to produce the substrates or obtain them from the host by different transporters. The utilization of amino acid metabolizing pathways ultimately leads to formation of ammonia, a product known to be harmful for gut cells. On the other hand, obtaining essential amino acids (like Lysine) from the host is expected to create a competitive environment in the gut. Thus, the study emphasizes that it is not only important to know the butyrate producing capabilities of gut bacteria, but also the biosynthetic pathways used by them as well as the by-products formed in the process. In addition to gut pathogens, oral pathogens like *Porphyromonas, Filifactor*, and *Tannerella* also show presence of butyrate production pathways. These bacteria have evolved to utilize Pyruvate as well as amino acid fermentation for butyrate production. As explained earlier, the fact that butyrate itself is pathogenic in oral environment probably does not lead to an evolutionary selection of specific butyrogenic pathways for these pathogens. This observation is contrary to what is observed in case of gut pathogens as they have acquired harmful ammonia releasing amino acid metabolizing pathways for butyrate production.

The present study thus illustrates that commensals and pathogens might have evolved to harbor different pathways for biosynthesizing the same product. In addition, the different roles of a metabolite in specific body sites within a host might also influence distribution and evolution of butyrogenic pathways in bacteria. The findings from the current analyses can potentially be applied in future for manipulating butyrate production in order to improve enteric as well as oral health.

## Materials and Methods

### Genome Mining and Prediction of Butyrate Biosynthesis Pathways in Bacteria

Firstly, it was important to confirm that butyrate biosynthesizing genes occur in context with each other on the genome in a conserved arrangement. A HMM based analysis (Eddy, 1998) was performed on the genomes of bacteria known to produce butyrate through experimental studies. The HMMs corresponding to each of the domains within genes associated with the four butyrate production pathways (**Figure 2**) were obtained from the PFAM database (Finn et al., 2014). The genomes were scanned for butyrate pathways using hmmscan program from HMMER v. 3.1 (Eddy, 1998) with an *e*-value cutoff of 1e-06. In-house perl scripts were used to extract domains belonging to each pathway and their positions on corresponding genomes were obtained using PTT files. This information was utilized for gene context analysis. In-house scripts were used to extract the hits to the HMM where all genes (domains) within a pathway were not only present, but occurred consecutively (within five genes from each other) on the genomes. In addition, the order of occurrence of these domains was noted for each genome.

After confirming the gene arrangement for all the four pathways in bacteria mentioned above, the analysis was extended to other bacterial genomes. Protein sequences (FASTA and ptt files) corresponding to bacterial genomes (complete: 2749; draft: 5503) were downloaded from the NCBI^1^. The HMM protocol described above was applied to all genomes and a table cataloging the genes in each of the butyrate pathways present in these genomes was obtained (Supplementary Table S1).

### Identification of Butyrate Producers in Human Gut and Oral Cavities

In order to identify butyrogenic bacteria in human gut, publicly available datasets containing PCR amplicons for 16S rRNA corresponding to each of the following studies were downloaded from NCBI-SRA^2^.

• Gut microbiome of 30 healthy individuals and 30 Colorectal Cancer patients from Toronto (Canada), Boston (USA), Houston (USA), Ann Arbor (USA) (Zackular et al., 2014) (Fastq files available at http://www.mothur.org/MicrobiomeBiomarkerCRC).
• Gut microbiome of 56 healthy and 46 Colorectal Cancer subjects from Shanghai (China) (Wang et al., 2012) (SRA ID: SRP005150).
• Microbiome associated with biopsy samples of 95 Colorectal adenocarcinoma and their adjacent non-affected tissue from patients in Barcelona (Spain) (Kostic et al., 2012) (SRA ID: SRP000383).
• Gut microbiome of 302 IBD patients and 168 healthy individuals from African Caucasian population (Gevers et al., 2014) (Bioproject ID: PRJNA237362).
• Gut microbiome of 44 type II diabetes patients before and after (12 weeks) administration of a Chinese decoction (Xu et al., 2015).
• Gut microbiome of 50 obese and 50 non-obese individuals from Amish population (NCBI SRA ID SRX021087).
• Oral microbiome of 10 healthy individuals and 10 subgingival periodontitis patients from Chinese population (Tsai et al., 2016) (NCBI Bioproject ID PRJNA274944).
• Oral micriobiome of 25 healthy and 25 chronic periodontitis samples from American population (Kirst et al., 2015) (NCBI Bioproject ID PRJNA269205).

SRA toolkit version 2.3.4 was used to obtain fastq files from the downloaded data. Quality filtration of the 16S rRNA amplicons was performed to retain only those sequences that had an average phred score of more than 25. The quality filtered sequences were scanned to extract only the specific V-regions used in each of these studies using VXtractor 2.0 (Hartmann et al., 2010).

Taxonomic classification of sequences in each of the samples was performed using naïve Bayesian classifier implemented in the Ribosomal Database Project classifier (version 2.8) (Wang et al., 2007) executed at a bootstrap confidence cut-off of 80%. Based on reliability of alignments obtained at each taxonomic level, output sequences were classified at phylum, family and genus levels. The data was then normalized to obtain relative abundance at various taxonomic levels in each sample. Only the genera whose median relative abundance was greater than 0.001% for healthy or diseased datasets were retained for further analysis. Butyrate producers in the gut were then identified by mapping species belonging to each genus to butyrate production pathways listed in Supplementary Table S1.

### Comparison of Butyrate Production Pathways in Healthy and Dysbiotic Gut/Oral Cavities

In order to identify differentially abundant taxa in healthy and diseased cohorts, the obtained normalized taxa abundances in each of the datasets were subjected to multivariate data analysis. To remove sample outliers (based on the abundance values), *t*-test was performed with 1000 iterations of bootstrapping. In other words, 80% of the samples were randomly selected in each iteration from healthy as well as diseased datasets and a *t*-test was carried out on these samples. The genera that appeared as significantly different (in terms of abundance with a *p*-value < 0.05) in more than 50% of the iterations (500) were selected. The differential genera so obtained were analyzed further to see the differences in distribution of butyrogenic pathways between commensals and pathogens.

## Author Contributions

SA, HK, and SSM conceived and designed the experiments; SA and HK conducted the experiments; SA, HK, and SM analyzed the data; SA, HK, and SM wrote the manuscript; All authors discussed the results, commented on the manuscript and approved the final version. SA and HK have equally contributed to the manuscript.

## Conflict of Interest Statement

The authors declare that the research was conducted in the absence of any commercial or financial relationships that could be construed as a potential conflict of interest.

## Footnotes

1 ftp://ftp.ncbi.nlm.nih.gov/genomes/genbank/bacteria/

2 ftp://ftp.ncbi.nlm.nih.gov/sra
