# Supplementary material for: Comparative In silico Analysis of Butyrate Production Pathways in Gut Commensals and Pathogens
Source: Front Microbiol. 2016 Dec 2;7:1945. doi: 10.3389/fmicb.2016.01945 (PMC5133246; doi:10.3389/fmicb.2016.01945)
Supplement: Supplementary file 1 [file Data_Sheet_1.PDF]

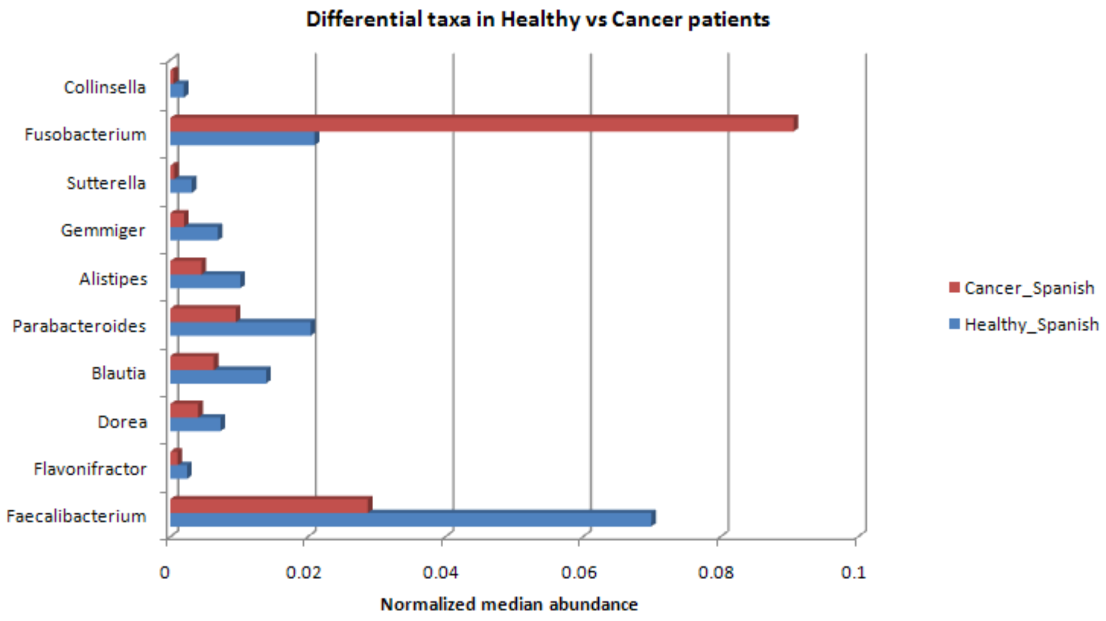

Differential taxa in Healthy vs Cancer patients
Collinsella
Fusobacterium
Sutterella
Gemmiger
Alistipes
Parabacteroides
Blautia
Dorea
Flavonifractor
Faecalibacterium
0
0.02
0.04
0.06
0.08
0.1
Normalized median abundance
Cancer_Spanish
Healthy_Spanish

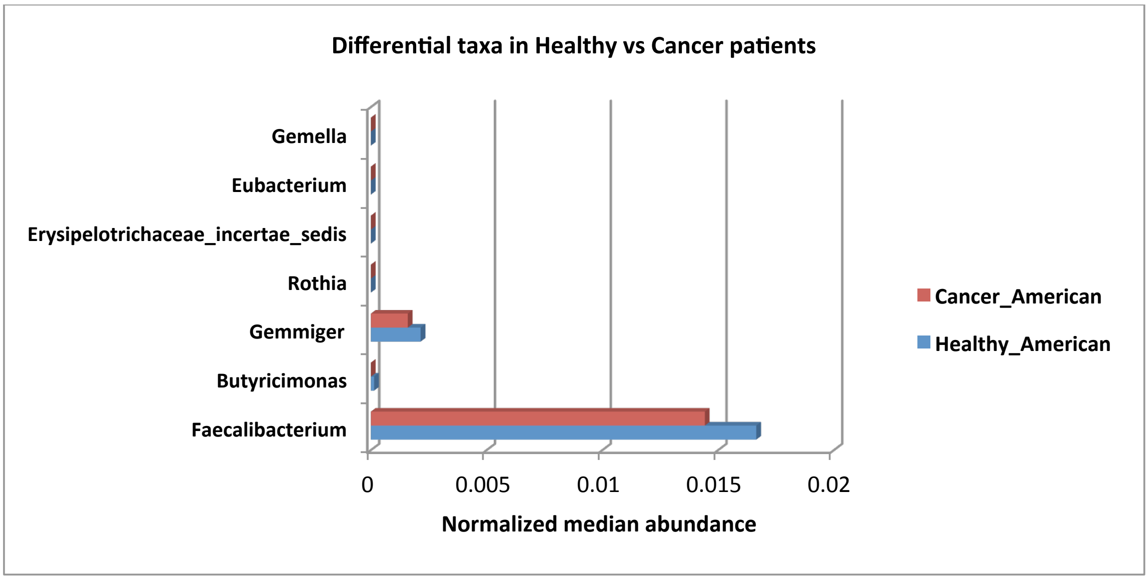

Differential taxa in Healthy vs Cancer patients
Gemella
Eubacterium
Erysipelotrichaceae_incertae_sedis
Rothia
Gemmiger
Butyricimonas
Faecalibacterium
0
0.005
0.01
0.015
0.02
Normalized median abundance
Cancer_American
Healthy_American

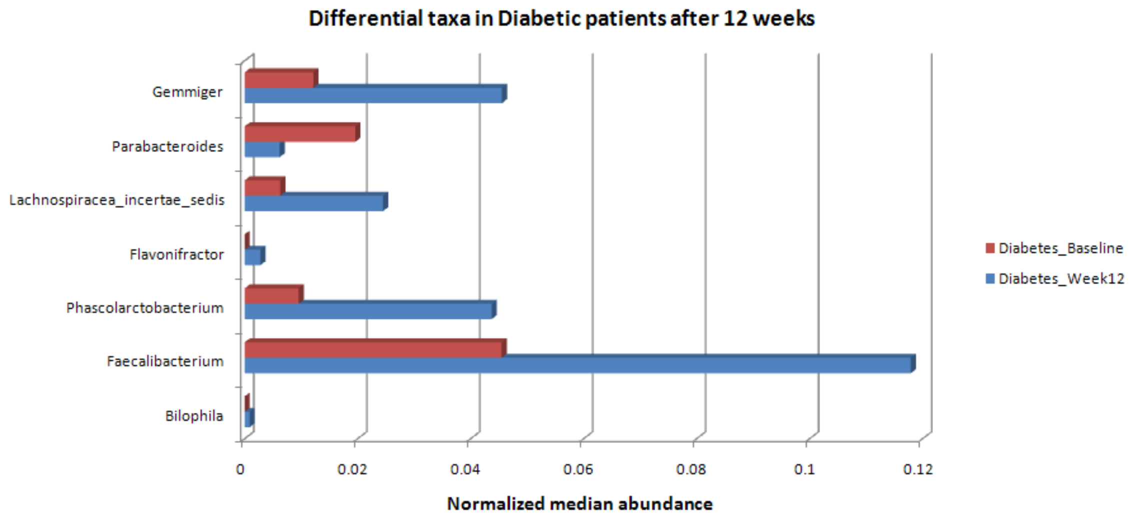
Differential taxa in Diabetic patients after 12 weeks
Gemmiger
Parabacteroides
Lachnospiracea_incertae_sedis
Flavonifractor
Phascolarctobacterium
Faecalibacterium
Bilophila
0
0.02
0.04
0.06
0.08
0.1
0.12
Normalized median abundance
Diabetes_Baseline
Diabetes_Week12

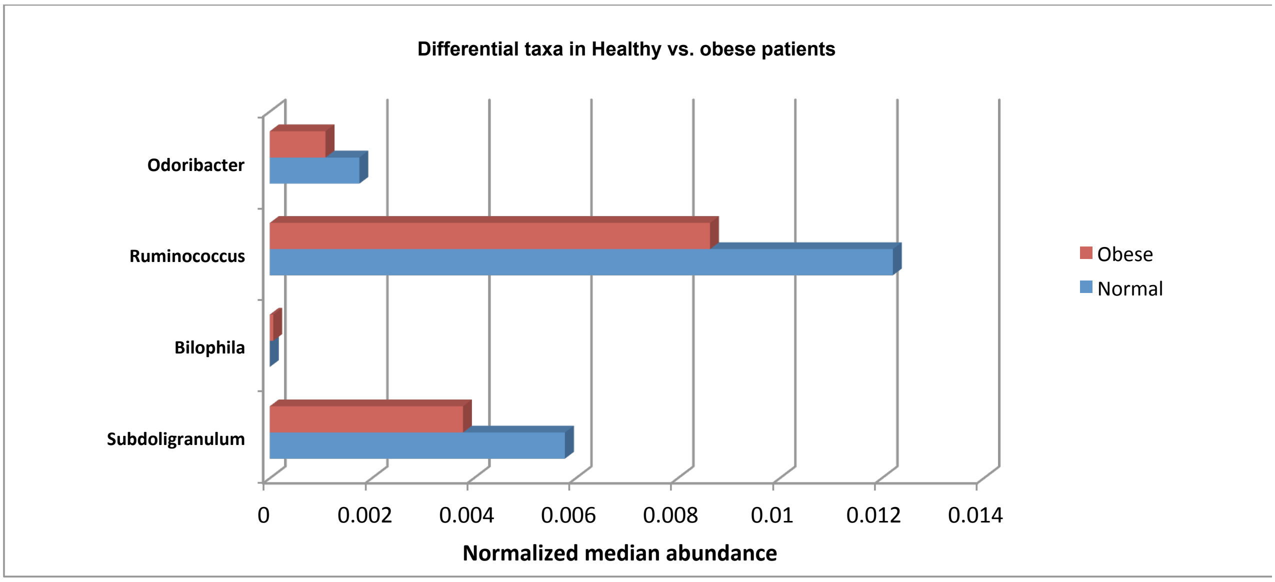
Differential taxa in Healthy vs. obese patients
Odoribacter
Ruminococcus
Bilophila
Subdoligranulum
0
0.002
0.004
0.006
0.008
0.01
0.012
0.014
Normalized median abundance
Obese
Normal

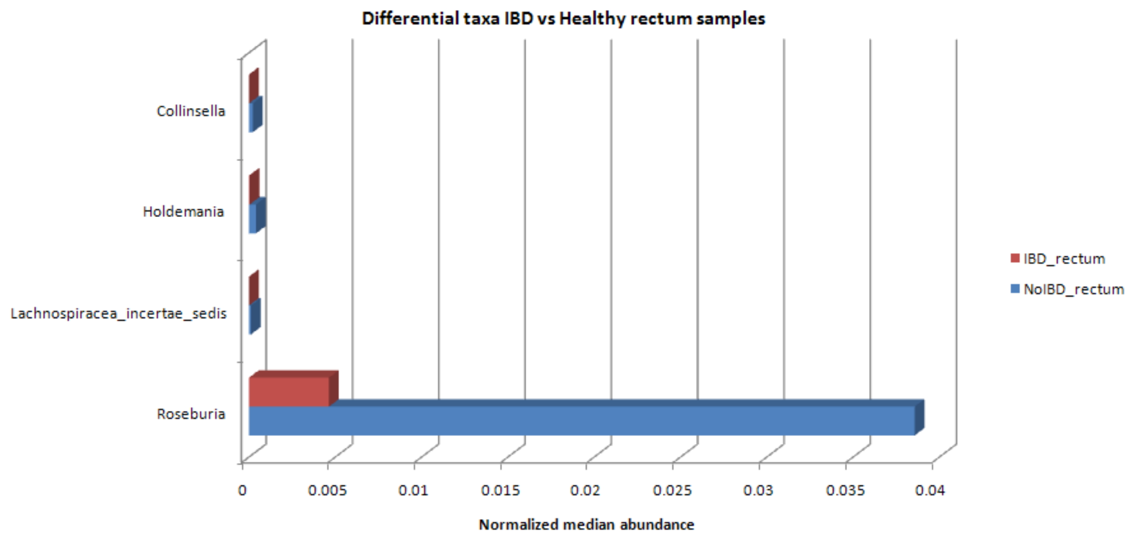
Differential taxa IBD vs Healthy rectum samples
Collinsella
Holdemania
Lachnospiracea_incertae_sedis
Roseburia
0
0.005
0.01
0.015
0.02
0.025
0.03
0.035
0.04
Normalized median abundance
IBD_rectum
NoIBD_rectum

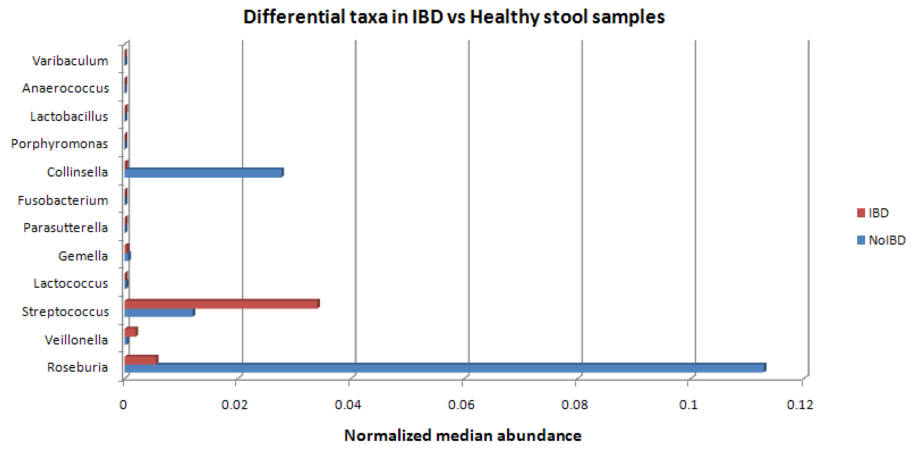
Differential taxa in IBD vs Healthy stool samples
Varibaculum
Anaerococcus
Lactobacillus
Porphyromonas
Collinsella
Fusobacterium
Parasutterella
Gemella
Lactococcus
Streptococcus
Veillonella
Roseburia
0
0.02
0.04
0.06
0.08
0.1
0.12
Normalized median abundance
IBD
NoIBD

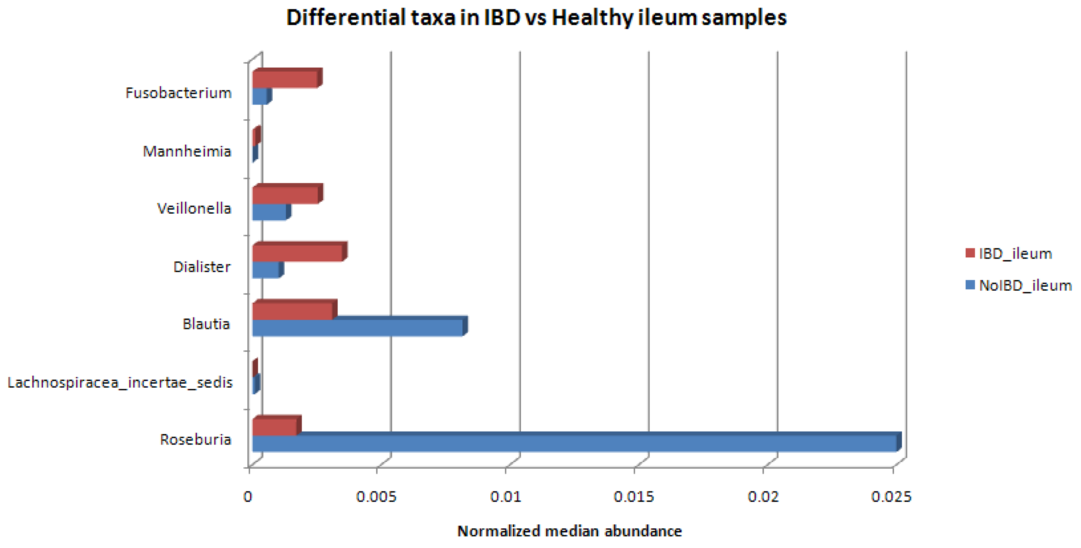
Differential taxa in IBD vs Healthy ileum samples
Fusobacterium
Mannheimia
Veillonella
Dialister
Blautia
Lachnospiracea_incertae_sedis
Roseburia
0
0.005
0.01
0.015
0.02
0.025
Normalized median abundance
IBD_ileum
NoIBD_ileum

Figure S1

Differentially abundant taxa in healthy vs diseased samples in various datasets.

Top to bottom:

Healthy vs. Cancer Spanish CRC samples, Healthy vs. Cancer American CRC samples, Healthy vs. Diabetic Chinese population, Healthy vs Obese Amish population and Healthy vs IBD patients-ileum, stool and rectum samples.

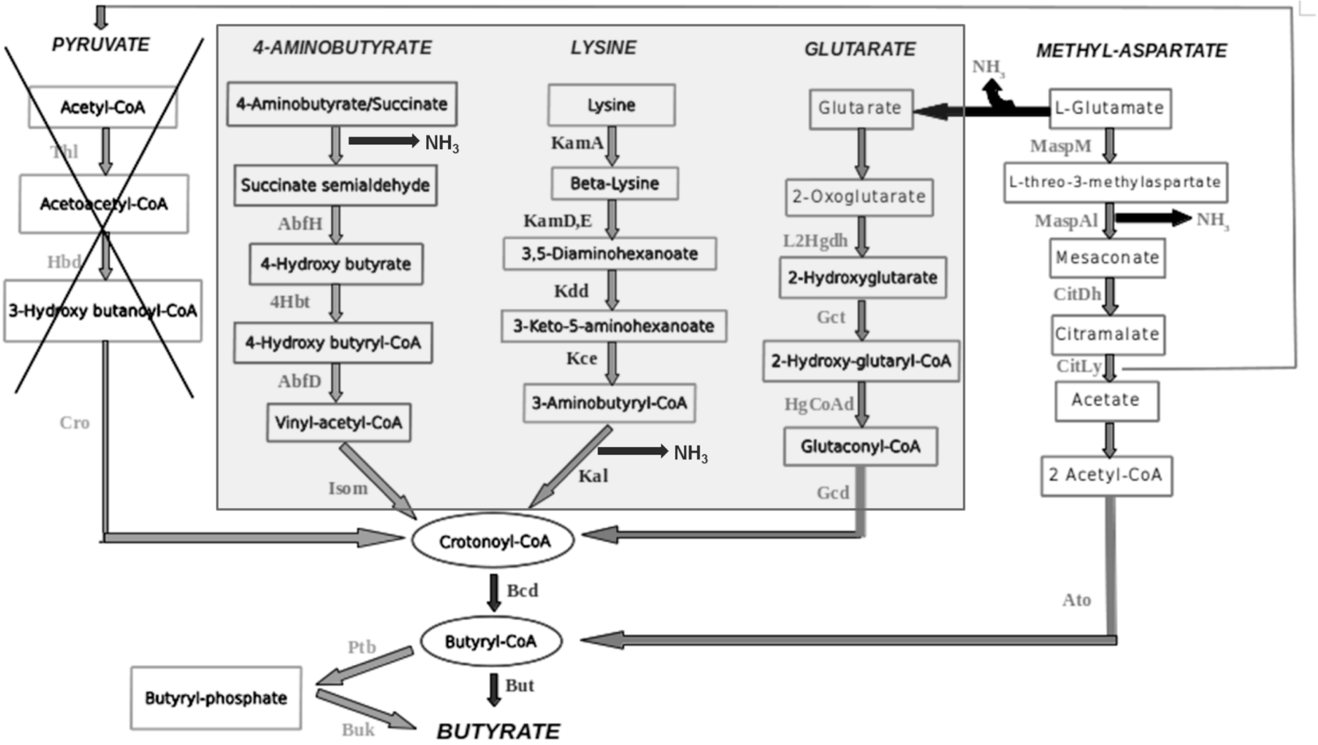

Supplementary Fig. S2

Butyrate production pathway in *Fusobacteria*

Out of the four butyrate production pathways, three (shaded box) are observed in *Fusobacteria*. Methylaspartate pathway, an additional pathway for butyrate production, involves methylaspartate mutase (MaspM), methylaspartate ammonia lyase (MaspAl), Citramalate dehydratase (CitDh), Citramalate lyase (CitLy) and butyryl-CoA:acetoacetate CoA-transferase (Ato). All butyrate production pathways in *Fusobacteria* concomitantly release ammonia during amino acid metabolism.

Genomes	Pyruvate	4-aminobutyrate	Lysine	Glutarate
Achromobacter_xylosoxidans_A8_uid59899	1	0	0	0
Acidaminococcus_fermentans_DSM_20731_uid43471	1	0	0	1
Acidaminococcus_intestini_RyC_MR95_uid74445	1	0	0	1
Azospirillum_B510_uid46085	1	0	0	0
Azotobacter_vinelandii_CA6_uid198830	1	0	0	0
Azotobacter_vinelandii_CA_uid198829	1	0	0	0
Azotobacter_vinelandii_DJ_uid57597	1	0	0	0
Brachyspira_pilosicoli_95_1000_uid50609	1	0	0	0
Brachyspira_pilosicoli_B2904_uid175255	1	0	0	0
Brachyspira_pilosicoli_P43_6_78_uid184077	1	0	0	0
Brachyspira_pilosicoli_WesB_uid175256	1	0	0	0
Butyrivibrio_fibrisolvens_uid197155	1	0	0	0
Butyrivibrio_proteoclasticus_B316_uid51489	1	0	0	0
Clostridium_botulinum_Ba4_657_uid59173	1	0	0	0
Clostridium_botulinum_BKT015925_uid66203	1	0	0	0
Clostridium_botulinum_F_230613_uid159513	1	0	0	0
Clostridium_botulinum_H04402_065_uid162091	1	0	0	0
Clostridium_cellulovorans_743B_uid51503	1	0	0	0
Clostridium_cf__saccharolyticum_K10_uid197201	1	0	0	1
Clostridium_difficile_630_uid57679	1	1	0	0
Clostridium_difficile_BI1_uid158363	1	1	0	0
Clostridium_difficile_CD196_uid41017	1	1	0	0
Clostridium_difficile_R20291_uid40921	1	1	0	0
Clostridium_kluyveri_DSM_555_uid58885	1	1	0	0
Clostridium_kluyveri_NBRC_12016_uid59369	1	1	0	0
Clostridium_pasteurianum_BC1_uid201478	1	0	0	0
Clostridium_perfringens_13_uid57681	1	0	0	0
Clostridium_saccharobutylicum_DSM_13864_uid223284	1	0	0	0
Clostridium_saccharoperbutylacetonicum_ATCC_27021_uid189747	1	0	0	0
Clostridium_tetani_12124569_uid227214	1	0	0	0
Clostridium_tetani_E88_uid57683	1	0	0	0
Deferribacter_desulfuricans_SSM1_uid46653	1	0	0	0
Desulfitobacterium_dehalogenans_ATCC_51507_uid82553	1	0	0	0
Desulfitobacterium_dichloroeliminans_LMG_P_21439_uid82555	1	0	0	0
Desulfitobacterium_hafniense_DCB_2_uid57749	1	0	0	0
Desulfitobacterium_hafniense_Y51_uid58605	1	0	0	0
Desulfobulbus_propionicus_DSM_2032_uid62265	1	0	0	0
Desulfotomaculum_gibsoniae_DSM_7213_uid76945	1	0	0	0
Desulfotomaculum_kuznetsovii_DSM_6115_uid67357	1	0	0	0
Desulfotomaculum_reducens_MI_1_uid58277	1	0	0	0
Desulfurispirillum_indicum_S5_uid45897	1	0	0	0
Eubacterium_limosum_KIST612_uid59777	1	0	0	0
Eubacterium_rectale_ATCC_33656_uid59169	1	0	0	0
Eubacterium_rectale_uid197161	1	0	0	0
Eubacterium_rectale_uid197162	1	0	0	0
Faecalibacterium_prausnitzii_L2_6_uid197183	1	0	0	0
Faecalibacterium_prausnitzii_uid197157	1	0	0	0
Filifactor_alocis_ATCC_35896_uid46625	1	0	0	1
Halanaerobium_praevalens_DSM_2228_uid161959	1	0	1	0
Heliobacterium_modesticaldum_Ice1_uid58279	1	0	0	0
Leptothrix_cholodnii_SP_6_uid58971	1	0	0	0
Odoribacter_splanchnicus_DSM_20712_uid63397	1	1	0	0
Oscillibacter_valericigenes_uid73895	1	0	0	1
Porphyromonas_asaccharolytica_DSM_20707_uid66603	1	1	1	0
Porphyromonas_gingivalis_ATCC_33277_uid58879	1	1	1	0
Porphyromonas_gingivalis_TDC60_uid67407	1	1	1	0
Porphyromonas_gingivalis_W83_uid57641	1	1	1	0
Roseburia_hominis_A2_183_uid73419	1	0	0	0
Roseburia_intestinalis_uid197164	1	0	0	0
Thermoanaerobacterium_thermosaccharolyticum_DSM_571_uid51639	1	0	0	0
Thermoanaerobacterium_thermosaccharolyticum_M0795_uid184821	1	0	0	0
Thermoanaerobacter_tengcongensis_MB4_uid57813	1	0	1	0
Thermoanaerobacter_wiegelii_Rt8_B1_uid52581	0	0	1	0
Deinococcus_gobiensis_I_0_uid162509	0	1	0	0
Megasphaera_elsdenii_DSM_20460_uid71135	0	1	0	0
Tannerella_forsythia_ATCC_43037_uid83157	0	1	0	0
Actinoplanes_friuliensis_DSM_7358_uid226110	0	0	1	0
Actinoplanes_missouriensis_431_uid158169	0	0	1	0
Actinoplanes_N902_109_uid202219	0	0	1	0
Actinoplanes_SE50_110_uid162333	0	0	1	0

Alkaliphilus_metalliredigens_QYMF_uid58171	0	0	1	0
Alkaliphilus_oremlandii_OhILAs_uid58495	0	0	1	0
Anaeromyxobacter_dehalogenans_2CP_1_uid58989	0	0	1	0
Anaeromyxobacter_dehalogenans_2CP_C_uid58135	0	0	1	0
Anaeromyxobacter_Fw109_5_uid58755	0	0	1	0
Anaeromyxobacter_K_uid58953	0	0	1	0
Azoarcus_KH32C_uid193704	0	0	1	0
Bacteroides_CF50_uid222805	0	0	1	0
Clostridium_SY8519_uid68705	0	0	1	0
Corallococcus_coralloides_DSM_2259_uid157997	0	0	1	0
Desulfosporosinus_acidiphilus_SJ4_uid156759	0	0	1	0
Fervidobacterium_nodosum_Rt17_B1_uid58625	0	0	1	0
Fervidobacterium_pennivorans_DSM_9078_uid78143	0	0	1	0
Fusobacterium_3_1_36A2_uid55995	0	0	1	1
Fusobacterium_4_8_uid205051	0	0	1	1
Fusobacterium_nucleatum_ATCC_25586_uid57885	0	0	1	1
Fusobacterium_nucleatum_polymorphum_ATCC_10953_uid54419	0	0	1	1
Kosmotoga_olearia_TBF_19_5_1_uid59205	0	0	1	0
Marinitoga_piezophila_KA3_uid81629	0	0	1	0
Mesotoga_prima_MesG1_Ag_4_2_uid52599	0	0	1	0
Myxococcus_fulvus_HW_1_uid68443	0	0	1	0
Myxococcus_stipitatus_DSM_14675_uid186549	0	0	1	0
Natranaerobius_thermophilus_JW_NM_WN_LF_uid59001	0	0	1	0
Petrotoga_mobilis_SJ95_uid58747	0	0	1	0
Rhodoferax_ferrireducens_T118_uid58353	0	0	1	0
Saccharothrix_espanaensis_DSM_44229_uid184826	0	0	1	0
Salinispora_tropica_CNB_440_uid58565	0	0	1	0
Stigmatella_aurantiaca_DW4_3_1_uid158509	0	0	1	0
Symbiobacterium_thermophilum_IAM_14863_uid58165	0	0	1	0
Thermosipho_africanus_TCF52B_uid59095	0	0	1	0

Table S1

The table contains a catalogue of all sequenced butyrate producing bacterial genomes and corresponding pathways for butyrate production.

Genomes	Pyruvate	4-aminobutyrate	Lysine	Glutarate
Acaryochloris_marina_MBIC11017_uid58167	1	0	0	0
Achromobacter_xylosoxidans_NBRC_15126_uid232243	1	0	0	0
Achromobacter_xylosoxidans_uid205255	1	0	0	0
Acidilobus_saccharovorans_345_15_uid51395	1	0	0	0
Acidimicrobium_ferrooxidans_DSM_10331_uid59215	1	0	0	0
Acidiphilium_cryptum_JF_5_uid58447	1	0	0	0
Acidiphilium_multivorum_AIU301_uid63345	1	0	0	0
Acidobacterium_MP5ACTX9_uid50551	1	0	0	0
Acidothermus_cellulolyticus_11B_uid58501	1	0	0	0
Acidovorax_avenae_ATCC_19860_uid42497	1	0	0	0
Acidovorax_citrulli_AAC00_1_uid58429	1	0	0	0
Acidovorax_ebreus_TPSY_uid59233	1	0	0	0
Acidovorax_JS42_uid58427	1	0	0	0
Acidovorax_KKS102_uid176500	1	0	0	0
Acinetobacter_ADP1_uid61597	1	0	0	0
Acinetobacter_baumannii_1656_2_uid158677	1	0	0	0
Acinetobacter_baumannii_AB0057_uid59083	1	0	0	0
Acinetobacter_baumannii_AB307_0294_uid59271	1	0	0	0
Acinetobacter_baumannii_ATCC_17978_uid58731	1	0	0	0
Acinetobacter_baumannii_AYE_uid61637	1	0	0	0
Acinetobacter_baumannii_BJAB07104_uid210971	1	0	0	0
Acinetobacter_baumannii_BJAB0715_uid210972	1	0	0	0
Acinetobacter_baumannii_BJAB0868_uid210973	1	0	0	0
Acinetobacter_baumannii_MDR_TJ_uid162739	1	0	0	0
Acinetobacter_baumannii_TCDC_AB0715_uid158679	1	0	0	0
Acinetobacter_baumannii_TYTH_1_uid176498	1	0	0	0
Acinetobacter_baumannii_ZW85_1_uid231518	1	0	0	0
Acinetobacter_calcoaceticus_PHEA_2_uid83123	1	0	0	0
Acinetobacter_oleivorans_DR1_uid50119	1	0	0	0
Actinosynnema_mirum_DSM_43827_uid58951	1	0	0	0
Advenella_kashmirensis_WT001_uid80859	1	0	0	0
Aequorivita_sublithincola_DSM_14238_uid168181	1	0	0	0
Aeromonas_hydrophila_ML09_119_uid205540	1	0	0	0
Aeromonas_salmonicida_A449_uid58631	1	0	0	0
Agrobacterium_fabrum_C58_uid57865	1	0	0	0
Agrobacterium_H13_3_uid63403	1	0	0	0
Agrobacterium_radiobacter_K84_uid58269	1	0	0	0
Agrobacterium_vitis_S4_uid58249	1	0	0	0
Agromonas_oligotrophica_S58_uid192186	1	0	0	0
Alcanivorax_borkumensis_SK2_uid58169	1	0	0	0
Alcanivorax_dieselolei_B5_uid176364	1	0	0	0
Alicycliphilus_denitrificans_BC_uid49953	1	0	0	0
Alicycliphilus_denitrificans_K601_uid66307	1	0	0	0
Alicyclobacillus_acidocaldarius_DSM_446_uid59199	1	0	0	0
Alicyclobacillus_acidocaldarius_Tc_4_1_uid158681	1	0	0	0
Alkalilimnicola_ehrlichii_MLHE_1_uid58467	1	0	0	0
Alteromonas_macleodii__Aegean_Sea_MED64__uid2316[illegible]	1	0	0	0
Alteromonas_macleodii_AltDE1_uid179068	1	0	0	0
Alteromonas_macleodii_ATCC_27126_uid55253	1	0	0	0
Alteromonas_macleodii__Balearic_Sea_AD45__uid17636[illegible]	1	0	0	0
Alteromonas_macleodii__Black_Sea_11__uid176365	1	0	0	0
Alteromonas_macleodii__Deep_ecotype__uid58251	1	0	0	0
Alteromonas_macleodii__English_Channel_615__uid2107[illegible]	1	0	0	0
Alteromonas_macleodii__English_Channel_673__uid1763[illegible]	1	0	0	0
Alteromonas_macleodii__Ionian_Sea_U4__uid210780	1	0	0	0
Alteromonas_macleodii__Ionian_Sea_U7__uid210785	1	0	0	0
Alteromonas_macleodii__Ionian_Sea_U8__uid210782	1	0	0	0
Alteromonas_macleodii__Ionian_Sea_UM4b__uid210784	1	0	0	0
Alteromonas_macleodii__Ionian_Sea_UM7__uid210783	1	0	0	0
Alteromonas_SN2_uid67349	1	0	0	0
Amycolatopsis_mediterranei_RB_uid216089	1	0	0	0
Amycolatopsis_mediterranei_S699_uid158689	1	0	0	0
Amycolatopsis_mediterranei_S699_uid171830	1	0	0	0
Amycolatopsis_mediterranei_U32_uid50565	1	0	0	0
Amycolatopsis_orientalis_HCCB10007_uid203791	1	0	0	0
Amycolicicoccus_subflavus_DQS3_9A1_uid67253	1	0	0	0
Anoxybacillus_flavithermus_WK1_uid59135	1	0	0	0
Archaeoglobus_fulgidus_DSM_4304_uid57717	1	0	0	0
Archaeoglobus_sulfaticallidus_PM70_1_uid201033	1	0	0	0

Arcobacter_L_uid158135	1	0	0	0
Arcobacter_nitrofigilis_DSM_7299_uid49001	1	0	0	0
Aromatoleum_aromaticum_EbN1_uid58231	1	0	0	0
Azoarcus_BH72_uid61603	1	0	0	0
Azorhizobium_caulinodans_ORS_571_uid58905	1	0	0	0
Azospirillum_brasilense_Sp245_uid162161	1	0	0	0
Azospirillum_lipoferum_4B_uid82343	1	0	0	0
Bacillus_1NLA3E_uid81841	1	0	0	0
Bacillus_amyloliquefaciens_CC178_uid226115	1	0	0	0
Bacillus_amyloliquefaciens_DSM_7_uid53535	1	0	0	0
Bacillus_amyloliquefaciens_FZB42_uid58271	1	0	0	0
Bacillus_amyloliquefaciens_IT_45_uid181617	1	0	0	0
Bacillus_amyloliquefaciens_LFB112_uid232246	1	0	0	0
Bacillus_amyloliquefaciens_LL3_uid158133	1	0	0	0
Bacillus_amyloliquefaciens_plantarum_AS43_3_uid183682	1	0	0	0
Bacillus_amyloliquefaciens_plantarum_CAU_B946_uid842	1	0	0	0
Bacillus_amyloliquefaciens_plantarum_NAU_B3_uid22281	1	0	0	0
Bacillus_amyloliquefaciens_plantarum_UCMB5033_uid215	1	0	0	0
Bacillus_amyloliquefaciens_plantarum_UCMB5036_uid190	1	0	0	0
Bacillus_amyloliquefaciens_plantarum_UCMB5113_uid215	1	0	0	0
Bacillus_amyloliquefaciens_plantarum_YAU_B9601_Y2_u	1	0	0	0
Bacillus_amyloliquefaciens_TA208_uid158701	1	0	0	0
Bacillus_amyloliquefaciens_XH7_uid158881	1	0	0	0
Bacillus_amyloliquefaciens_Y2_uid165195	1	0	0	0
Bacillus_anthracis_A2012_uid54101	1	0	0	0
Bacillus_anthracis__Ames_Ancestor__uid58083	1	0	0	0
Bacillus_anthracis_Ames_uid57909	1	0	0	0
Bacillus_anthracis_CDC_684_uid59303	1	0	0	0
Bacillus_anthracis_H9401_uid162021	1	0	0	0
Bacillus_anthracis_Sterne_uid58091	1	0	0	0
Bacillus_atrophaeus_1942_uid59887	1	0	0	0
Bacillus_cellulosilyticus_DSM_2522_uid43329	1	0	0	0
Bacillus_cereus_03BB102_uid59299	1	0	0	0
Bacillus_cereus_AH187_uid58753	1	0	0	0
Bacillus_cereus_AH820_uid58751	1	0	0	0
Bacillus_cereus_ATCC_10987_uid57673	1	0	0	0
Bacillus_cereus_ATCC_14579_uid57975	1	0	0	0
Bacillus_cereus_biovar_anthracis_CI_uid50615	1	0	0	0
Bacillus_cereus_E33L_uid58103	1	0	0	0
Bacillus_cereus_F837_76_uid83611	1	0	0	0
Bacillus_cereus_FRI_35_uid173403	1	0	0	0
Bacillus_cereus_G9842_uid58759	1	0	0	0
Bacillus_cereus_NC7401_uid82815	1	0	0	0
Bacillus_cereus_Q1_uid58529	1	0	0	0
Bacillus_clausii_KSM_K16_uid58237	1	0	0	0
Bacillus_coagulans_2_6_uid68053	1	0	0	0
Bacillus_coagulans_36D1_uid54335	1	0	0	0
Bacillus_cytotoxicus_NVH_391_98_uid58317	1	0	0	0
Bacillus_halodurans_C_125_uid57791	1	0	0	0
Bacillus_infantis_NRRL_B_14911_uid222804	1	0	0	0
Bacillus_JS_uid162189	1	0	0	0
Bacillus_licheniformis_9945A_uid207072	1	0	0	0
Bacillus_licheniformis_ATCC_14580_uid58097	1	0	0	0
Bacillus_licheniformis_DSM_13___ATCC_14580_uid58199	1	0	0	0
Bacillus_megaterium_DSM319_uid48371	1	0	0	0
Bacillus_megaterium_QM_B1551_uid15862	1	0	0	0
Bacillus_megaterium_WSH_002_uid159841	1	0	0	0
Bacillus_pseudofirmus_OF4_uid45847	1	0	0	0
Bacillus_pumilus_SAFR_032_uid59017	1	0	0	0
Bacillus_selenitireducens_MLS10_uid49513	1	0	0	0
Bacillus_subtilis_168_uid57675	1	0	0	0
Bacillus_subtilis_6051_HGW_uid193706	1	0	0	0
Bacillus_subtilis_BAB_1_uid195461	1	0	0	0
Bacillus_subtilis_BSn5_uid62463	1	0	0	0
Bacillus_subtilis_BSP1_uid184010	1	0	0	0
Bacillus_subtilis_natto_BEST195_uid183001	1	0	0	0
Bacillus_subtilis_PY79_uid229877	1	0	0	0
Bacillus_subtilis_QB928_uid173926	1	0	0	0
Bacillus_subtilis_RO_NN_1_uid158879	1	0	0	0
Bacillus_subtilis_spizizenii_TU_B_10_uid73967	1	0	0	0

Bacillus_subtilis_spizizenii_W23_uid51879	1	0	0	0
Bacillus_subtilis_XF_1_uid189187	1	0	0	0
Bacillus_thuringiensis_Al_Hakam_uid58795	1	0	0	0
Bacillus_thuringiensis_BMB171_uid49135	1	0	0	0
Bacillus_thuringiensis_Bt407_uid177931	1	0	0	0
Bacillus_thuringiensis_HD_771_uid173374	1	0	0	0
Bacillus_thuringiensis_HD_789_uid173860	1	0	0	0
Bacillus_thuringiensis_MC28_uid176369	1	0	0	0
Bacillus_thuringiensis_serovar_chinensis_CT_43_uid1581	1	0	0	0
Bacillus_thuringiensis_serovar_finitimus_YBT_020_uid158	1	0	0	0
Bacillus_thuringiensis_serovar_IS5056_uid190186	1	0	0	0
Bacillus_thuringiensis_serovar_kurstaki_HD73_uid189188	1	0	0	0
Bacillus_thuringiensis_YBT_1518_uid229419	1	0	0	0
Bacillus_toyonensis_BCT_7112_uid227218	1	0	0	0
Bacillus_weihenstephanensis_KBAB4_uid58315	1	0	0	0
Bacteriovorax_marinus_SJ_uid82341	1	0	0	0
Beijerinckia_indica_ATCC_9039_uid59057	1	0	0	0
Belliella_baltica_DSM_15883_uid168182	1	0	0	0
Blastococcus_saxobsidens_DD2_uid89391	1	0	0	0
Bordetella_avium_197N_uid61563	1	0	0	0
Bordetella_bronchiseptica_253_uid178913	1	0	0	0
Bordetella_bronchiseptica_MO149_uid177517	1	0	0	0
Bordetella_bronchiseptica_RB50_uid57613	1	0	0	0
Bordetella_parapertussis_12822_uid57615	1	0	0	0
Bordetella_parapertussis_18323_uid175569	1	0	0	0
Bordetella_parapertussis_Bpp5_uid177516	1	0	0	0
Bordetella_pertussis_CS_uid158859	1	0	0	0
Bordetella_pertussis_Tohama_I_uid57617	1	0	0	0
Bordetella_petrii_uid61631	1	0	0	0
Bradyrhizobium_BTAi1_uid58505	1	0	0	0
Bradyrhizobium_japonicum_USDA_110_uid57599	1	0	0	0
Bradyrhizobium_japonicum_USDA_6_uid158851	1	0	0	0
Bradyrhizobium_ORS_278_uid58941	1	0	0	0
Bradyrhizobium_S23321_uid158167	1	0	0	0
Brevibacillus_brevis_NBRC_100599_uid59175	1	0	0	0
Brevundimonas_subvibrioides_ATCC_15264_uid42117	1	0	0	0
Brucella_abortus_A13334_uid83615	1	0	0	0
Brucella_abortus_bv__1_9_941_uid58019	1	0	0	0
Brucella_abortus_S19_uid58873	1	0	0	0
Brucella_canis_ATCC_23365_uid59009	1	0	0	0
Brucella_canis_HSK_A52141_uid83613	1	0	0	0
Brucella_ceti_TE10759_12_uid229880	1	0	0	0
Brucella_ceti_TE28753_12_uid229879	1	0	0	0
Brucella_melitensis_ATCC_23457_uid59241	1	0	0	0
Brucella_melitensis_biovar_Abortus_2308_uid62937	1	0	0	0
Brucella_melitensis_bv__1_16M_uid57735	1	0	0	0
Brucella_melitensis_M28_uid158857	1	0	0	0
Brucella_melitensis_M5_90_uid158855	1	0	0	0
Brucella_melitensis_NI_uid158853	1	0	0	0
Brucella_microti_CCM_4915_uid59319	1	0	0	0
Brucella_ovis_ATCC_25840_uid58113	1	0	0	0
Brucella_pinnipedialis_B2_94_uid71131	1	0	0	0
Brucella_suis_1330_uid159871	1	0	0	0
Brucella_suis_1330_uid57927	1	0	0	0
Brucella_suis_ATCC_23445_uid59015	1	0	0	0
Brucella_suis_VBI22_uid83617	1	0	0	0
Burkholderia_383_uid58073	1	0	0	0
Burkholderia_ambifaria_AMMD_uid58303	1	0	0	0
Burkholderia_ambifaria_MC40_6_uid58701	1	0	0	0
Burkholderia_CCGE1001_uid42975	1	0	0	0
Burkholderia_CCGE1002_uid42523	1	0	0	0
Burkholderia_CCGE1003_uid46253	1	0	0	0
Burkholderia_cenocepacia_AU_1054_uid58371	1	0	0	0
Burkholderia_cenocepacia_HI2424_uid58369	1	0	0	0
Burkholderia_cenocepacia_J2315_uid57953	1	0	0	0
Burkholderia_cenocepacia_MC0_3_uid58769	1	0	0	0
Burkholderia_cepacia_GG4_uid173858	1	0	0	0
Burkholderia_gladioli_BSR3_uid66301	1	0	0	0
Burkholderia_glumae_BGR1_uid59397	1	0	0	0
Burkholderia_KJ006_uid165871	1	0	0	0

Burkholderia_mallei_ATCC_23344_uid57725	1	0	0	0
Burkholderia_mallei_SAVP1_uid58387	1	0	0	0
Burkholderia_multivorans_ATCC_17616_uid58697	1	0	0	0
Burkholderia_multivorans_ATCC_17616_uid58909	1	0	0	0
Burkholderia_phenoliruptrix_BR3459a_uid176370	1	0	0	0
Burkholderia_phymatum_STM815_uid58699	1	0	0	0
Burkholderia_phytofirmans_PsJN_uid58729	1	0	0	0
Burkholderia_pseudomallei_1026b_uid162511	1	0	0	0
Burkholderia_pseudomallei_668_uid58389	1	0	0	0
Burkholderia_pseudomallei_BPC006_uid174460	1	0	0	0
Burkholderia_pseudomallei_K96243_uid57733	1	0	0	0
Burkholderia_pseudomallei_MSHR346_uid55259	1	0	0	0
Burkholderia_rhizoxinica_HKI_454_uid60487	1	0	0	0
Burkholderia_RPE64_uid205541	1	0	0	0
Burkholderia_vietnamiensis_G4_uid58075	1	0	0	0
Burkholderia_xenovorans_LB400_uid57823	1	0	0	0
Burkholderia_YI23_uid81081	1	0	0	0
Caldisphaera_lagunensis_DSM_15908_uid183486	1	0	0	0
Calditerrivibrio_nitroreducens_DSM_19672_uid60821	1	0	0	0
Candidatus_Accumulibacter_phosphatis_clade_IIA_UW_1	1	0	0	0
Candidatus_Chloracidobacterium_thermophilum_B_uid735	1	0	0	0
Candidatus_Koribacter_versatilis_Ellin345_uid58479	1	0	0	0
Candidatus_Methylomirabilis_oxyfera_uid161981	1	0	0	0
Candidatus_Nitrospira_defluvii_uid51175	1	0	0	0
Candidatus_Puniceispirillum_marinum_IMCC1322_uid470	1	0	0	0
Candidatus_Solibacter_usitatus_Ellin6076_uid58139	1	0	0	0
Carnobacterium_17_4_uid65789	1	0	0	0
Catenulispora_acidiphila_DSM_44928_uid59077	1	0	0	0
Caulobacter_crescentus_CB15_uid57891	1	0	0	0
Caulobacter_crescentus_NA1000_uid59307	1	0	0	0
Caulobacter_K31_uid58551	1	0	0	0
Caulobacter_segnis_ATCC_21756_uid41709	1	0	0	0
Cellulophaga_algicola_DSM_14237_uid62159	1	0	0	0
Cellulophaga_lytica_DSM_7489_uid63401	1	0	0	0
Chelativorans_BNC1_uid58069	1	0	0	0
Chromobacterium_violaceum_ATCC_12472_uid58001	1	0	0	0
Chroococcidiopsis_thermalis_PCC_7203_uid183002	1	0	0	0
Collimonas_fungivorans_Ter331_uid70793	1	0	0	0
Comamonas_testosteroni_CNB_2_uid62961	1	0	0	0
Conexibacter_woesei_DSM_14684_uid43467	1	0	0	0
Coprococcus_catus_GD_7_uid197174	1	0	0	0
Corynebacterium_argentoratense_DSM_44202_uid217419	1	0	0	0
Corynebacterium_aurimucosum_ATCC_700975_uid59409	1	0	0	0
Corynebacterium_efficiens_YS_314_uid62905	1	0	0	0
Corynebacterium_terpenotabidum_Y_11_uid210639	1	0	0	0
Coxiella_burnetii_CbuG_Q212_uid58893	1	0	0	0
Coxiella_burnetii_CbuK_Q154_uid58895	1	0	0	0
Coxiella_burnetii_RSA_493_uid57631	1	0	0	0
Croceibacter_atlanticus_HTCC2559_uid49661	1	0	0	0
Cupriavidus_metallidurans_CH34_uid57815	1	0	0	0
Cupriavidus_necator_N_1_uid68689	1	0	1	0
Cupriavidus_taiwanensis_LMG_19424_uid61615	1	0	1	0
Cyanothece_PCC_7425_uid59435	1	0	0	0
Cyclobacterium_marinum_DSM_745_uid71485	1	0	0	0
Cycloclasticus_P1_uid176368	1	0	0	0
Cycloclasticus_zancles_7_ME_uid214092	1	0	0	0
Cylindrospermum_stagnale_PCC_7417_uid183111	1	0	0	0
Cytophaga_hutchinsonii_ATCC_33406_uid57651	1	0	0	0
Dechloromonas_aromatica_RCB_uid58025	1	0	0	0
Dechlorosoma_suillum_PS_uid81439	1	0	0	0
Deinococcus_peraridilitoris_DSM_19664_uid183485	1	0	0	0
Delftia_acidovorans_SPH_1_uid58703	1	0	0	0
Delftia_Cs1_4_uid67319	1	0	0	0
Desulfarculus_baarsii_DSM_2075_uid51371	1	0	0	0
Desulfatibacillum_alkenivorans_AK_01_uid58913	1	0	0	0
Desulfobacterium_autotrophicum_HRM2_uid59061	1	0	0	0
Desulfomonile_tiedjei_DSM_6799_uid168320	1	0	0	0
Desulfosporosinus_meridiei_DSM_13257_uid75097	1	0	0	0
Desulfosporosinus_orientis_DSM_765_uid82939	1	0	0	0
Dinoroseobacter_shibae_DFL_12_uid58707	1	0	0	0

Echinicola_vietnamensis_DSM_17526_uid184076	1	0	0	0
Erythrobacter_litoralis_HTCC2594_uid58299	1	0	0	0
Exiguobacterium_antarcticum_B7_uid176125	1	0	0	0
Exiguobacterium_AT1b_uid59093	1	0	0	0
Exiguobacterium_MH3_uid227425	1	0	0	0
Exiguobacterium_sibiricum_255_15_uid58053	1	0	0	0
Ferrimonas_balearica_DSM_9799_uid53371	1	0	0	0
Ferroglobus_placidus_DSM_10642_uid40863	1	0	0	0
Ferroplasma_acidarmanus_fer1_uid54095	1	0	0	0
Flavobacteriaceae_bacterium_3519_10_uid59413	1	0	0	0
Flavobacteriales_bacterium_HTCC2170_uid51877	1	0	0	0
Flavobacterium_branchiophilum_FL_15_uid73421	1	0	0	0
Flavobacterium_columnare_ATCC_49512_uid80731	1	0	0	0
Flavobacterium_indicum_GPTSA100_9_uid157999	1	0	0	0
Flavobacterium_johnsoniae_UW101_uid58493	1	0	0	0
Flavobacterium_psychrophilum_JIP02_86_uid61627	1	0	0	0
Flexistipes_sinusarabici_DSM_4947_uid68147	1	0	0	0
Francisella_cf__novicida_3523_uid162107	1	0	0	0
Francisella_cf__novicida_Fx1_uid162105	1	0	0	0
Francisella_noatunensis_orientalis_LADL_07_285A_uid23	1	0	0	0
Francisella_noatunensis_orientalis_Toba_04_uid164779	1	0	0	0
Francisella_novicida_U112_uid58499	1	0	0	0
Francisella_philomiragia_ATCC_25017_uid59105	1	0	0	0
Francisella_tularensis_holarctica_FSC200_uid54341	1	0	0	0
Francisella_tularensis_holarctica_LVS_uid58595	1	0	0	0
Francisella_tularensis_mediasiatica_FSC147_uid58939	1	0	0	0
Francisella_tularensis_NE061598_uid161973	1	0	0	0
Francisella_tularensis_TI0902_uid89373	1	0	0	0
Francisella_tularensis_TIGB03_uid89379	1	0	0	0
Francisella_tularensis_WY96_3418_uid58811	1	0	0	0
Francisella_TX077308_uid68321	1	0	0	0
Frankia_alni_ACN14a_uid58695	1	0	0	0
Frankia_EAN1pec_uid58367	1	0	0	0
Frankia_EuI1c_uid42615	1	0	0	0
Frankia_symbiont_of_Datisca_glomerata_uid46257	1	0	0	0
Gallionella_capsiferriformans_ES_2_uid51505	1	0	0	0
Gemmatimonas_aurantiaca_T_27_uid58813	1	0	0	0
Geobacillus_C56_T3_uid49467	1	0	0	0
Geobacillus_HH01_uid188479	1	0	0	0
Geobacillus_JF8_uid215234	1	0	0	0
Geobacillus_kaustophilus_HTA426_uid58227	1	0	0	0
Geobacillus_thermodenitrificans_NG80_2_uid58829	1	0	0	0
Geobacillus_thermoglucosidasius_C56_YS93_uid48129	1	0	0	0
Geobacillus_thermoleovorans_CCB_US3_UF5_uid82949	1	0	0	0
Geobacillus_WCH70_uid59045	1	0	0	0
Geobacillus_Y412MC52_uid55381	1	0	0	0
Geobacillus_Y412MC61_uid41171	1	0	0	0
Geobacillus_Y4_1MC1_uid55779	1	0	0	0
Geobacter_bemidjiensis_Bem_uid58749	1	0	0	0
Geobacter_FRC_32_uid58543	1	0	0	0
Geobacter_M18_uid55771	1	0	0	0
Geobacter_M21_uid59037	1	0	0	0
Geobacter_metallireducens_GS_15_uid57731	1	0	0	0
Geobacter_uraniireducens_Rf4_uid58475	1	0	0	0
Glaciecola_4H_3_7_YE_5_uid66595	1	0	0	0
Glaciecola_nitratireducens_FR1064_uid73759	1	0	0	0
Glaciecola_psychrophila_170_uid193711	1	0	0	0
Gordonia_bronchialis_DSM_43247_uid41403	1	0	0	0
Gordonia_KTR9_uid174812	1	0	0	0
Gordonia_polyisoprenivorans_VH2_uid86651	1	0	0	0
Gramella_forsetii_KT0803_uid58881	1	0	0	0
Hahella_chejuensis_KCTC_2396_uid58483	1	0	0	0
Halalkalicoccus_jeotgali_B3_uid50305	1	0	0	0
Haliangium_ochraceum_DSM_14365_uid41425	1	0	0	0
Haloarcula_hispanica_ATCC_33960_uid72475	1	0	0	0
Haloarcula_hispanica_N601_uid230920	1	0	0	0
Haloarcula_marismortui_ATCC_43049_uid57719	1	0	0	0
Halobacillus_halophilus_DSM_2266_uid162033	1	0	0	0
Halobacterium_NRC_1_uid57769	1	0	0	0
Halobacterium_salinarum_R1_uid61571	1	0	0	0

Haloferax_mediterranei_ATCC_33500_uid167315	1	0	0	0
Haloferax_volcanii_DS2_uid46845	1	0	0	0
Halomicrobium_mukohataei_DSM_12286_uid59107	1	0	0	0
Halomonas_elongata_DSM_2581_uid52781	1	0	0	0
Halopiger_xanaduensis_SH_6_uid68105	1	0	0	0
Haloterrigena_turkmenica_DSM_5511_uid43501	1	0	0	0
Halovivax_ruber_XH_70_uid184819	1	0	0	0
Herbaspirillum_seropedicae_SmR1_uid50427	1	0	0	0
Herminiimonas_arsenicoxydans_uid58291	1	0	0	0
Herpetosiphon_aurantiacus_DSM_785_uid58599	1	0	0	0
Hirschia_baltica_ATCC_49814_uid59365	1	0	0	0
Hyphomicrobium_denitrificans_1NES1_uid179904	1	0	0	0
Hyphomicrobium_denitrificans_ATCC_51888_uid50325	1	0	0	0
Hyphomicrobium_MC1_uid68453	1	0	0	0
Hyphomicrobium_nitrativorans_NL23_uid230615	1	0	0	0
Idiomarina_loihiensis_GSL_199_uid205256	1	0	0	0
Idiomarina_loihiensis_L2TR_uid58087	1	0	0	0
Ilyobacter_polytropus_DSM_2926_uid59769	1	0	0	0
Intrasporangium_calvum_DSM_43043_uid61729	1	0	1	0
Jannaschia_CCS1_uid58147	1	0	0	0
Kangiella_koreensis_DSM_16069_uid59209	1	0	0	0
Kineococcus_radiotolerans_SRS30216_uid58067	1	0	0	0
Kitasatospora_setae_KM_6054_uid77027	1	0	0	0
Kocuria_rhizophila_DC2201_uid59099	1	0	0	0
Krokinobacter_4H_3_7_5_uid66593	1	0	0	0
Kyrpidia_tusciae_DSM_2912_uid48361	1	0	0	0
Lacinutrix_5H_3_7_4_uid68067	1	0	0	0
Laribacter_hongkongensis_HLHK9_uid59265	1	0	0	0
Legionella_longbeachae_NSW150_uid46099	1	0	0	0
Legionella_pneumophila_2300_99_Alcoy_uid48801	1	0	0	0
Legionella_pneumophila_ATCC_43290_uid86885	1	0	0	0
Legionella_pneumophila_Corby_uid58733	1	0	0	0
Legionella_pneumophila_Lens_uid58209	1	0	0	0
Legionella_pneumophila_Lorraine_uid170535	1	0	0	0
Legionella_pneumophila_Paris_uid58211	1	0	0	0
Legionella_pneumophila_Philadelphia_1_uid193710	1	0	0	0
Legionella_pneumophila_Philadelphia_1_uid57609	1	0	0	0
Legionella_pneumophila_Thunder_Bay_uid206517	1	0	0	0
Legionella_pneumophila_uid170534	1	0	0	0
Leisingera_methylohalidivorans_DSM_14336_uid232356	1	0	0	0
Leptolyngbya_PCC_7376_uid182928	1	0	0	0
Lysinibacillus_sphaericus_C3_41_uid58945	1	0	0	0
Magnetococcus_MC_1_uid57833	1	0	0	0
Magnetospirillum_magneticum_AMB_1_uid58527	1	0	0	0
Maricaulis_maris_MCS10_uid58689	1	0	0	0
Marinithermus_hydrothermalis_DSM_14884_uid65783	1	0	0	0
Marinobacter_adhaerens_HP15_uid162009	1	0	0	0
Marinobacter_aquaeolei_VT8_uid59419	1	0	0	0
Marinobacter_BSs20148_uid171995	1	0	0	0
Marinobacter_hydrocarbonoclasticus_ATCC_49840_uid16	1	0	0	0
Marinomonas_MWYL1_uid58715	1	0	0	0
Marivirga_tractuosa_DSM_4126_uid60837	1	0	0	0
Mesorhizobium_australicum_WSM2073_uid75101	1	0	0	0
Mesorhizobium_ciceri_biovar_biserrulae_WSM1271_uid62	1	0	0	0
Mesorhizobium_loti_MAFF303099_uid57601	1	0	0	0
Mesorhizobium_opportunistum_WSM2075_uid40861	1	0	0	0
Metallosphaera_cuprina_Ar_4_uid66329	1	0	0	0
Metallosphaera_sedula_DSM_5348_uid58717	1	0	0	0
Methylibium_petroleiphilum_PM1_uid58085	1	0	0	0
Methylobacterium_4_46_uid58843	1	0	0	0
Methylobacterium_chloromethanicum_CM4_uid58933	1	0	0	0
Methylobacterium_extorquens_AM1_uid57605	1	0	0	0
Methylobacterium_extorquens_DM4_uid61617	1	0	0	0
Methylobacterium_extorquens_PA1_uid58821	1	0	0	0
Methylobacterium_nodulans_ORS_2060_uid59023	1	0	0	0
Methylobacterium_populi_BJ001_uid58937	1	0	0	0
Methylobacterium_radiotolerans_JCM_2831_uid58845	1	0	0	0
Methylocella_silvestris_BL2_uid59433	1	0	0	0
Methylocystis_SC2_uid174072	1	0	0	0
Methylomicrobium_alcaliphilum_uid77119	1	0	0	0

Micrococcus_luteus_NCTC_2665_uid59033	1	0	0	0
Microcoleus_PCC_7113_uid183114	1	0	0	0
Micromonospora_aurantiaca_ATCC_27029_uid42501	1	0	0	0
Micromonospora_L5_uid45895	1	0	0	0
Muricauda_ruestringensis_DSM_13258_uid72479	1	0	0	0
Mycobacterium_abscessus_bolletii_50594_uid205422	1	0	0	0
Mycobacterium_abscessus_uid61613	1	0	0	0
Mycobacterium_avium_paratuberculosis_K_10_uid57699	1	0	0	0
Mycobacterium_avium_paratuberculosis_MAP4_uid20242(	1	0	0	0
Mycobacterium_chubuense_NBB4_uid168322	1	0	0	0
Mycobacterium_gilvum_PYR_GCK_uid59421	1	0	0	0
Mycobacterium_gilvum_Spyr1_uid61403	1	0	0	0
Mycobacterium_indicus_pranii_MTCC_9506_uid175523	1	0	0	0
Mycobacterium_intracellulare_ATCC_13950_uid167994	1	0	0	0
Mycobacterium_intracellulare_MOTT_02_uid89387	1	0	0	0
Mycobacterium_intracellulare_MOTT_64_uid89385	1	0	0	0
Mycobacterium_JDM601_uid67369	1	0	0	0
Mycobacterium_JLS_uid58489	1	0	0	0
Mycobacterium_kansasii_ATCC_12478_uid55385	1	0	0	0
Mycobacterium_KMS_uid58491	1	0	0	0
Mycobacterium_liflandii_128FXT_uid59005	1	0	0	0
Mycobacterium_marinum_M_uid59423	1	0	0	0
Mycobacterium_massiliense_GO_06_uid170732	1	0	0	0
Mycobacterium_MCS_uid58465	1	0	0	0
Mycobacterium_MOTT36Y_uid164001	1	0	0	0
Mycobacterium_rhodesiae_NBB3_uid75107	1	0	0	0
Mycobacterium_smegmatis_JS623_uid184820	1	0	0	0
Mycobacterium_smegmatis_MC2_155_uid171958	1	0	0	0
Mycobacterium_ulcerans_Agy99_uid62939	1	0	0	0
Mycobacterium_vanbaalenii_PYR_1_uid58463	1	0	0	0
Mycobacterium_VKM_Ac_1815D_uid199859	1	0	0	0
Mycobacterium_yongonense_05_1390_uid189649	1	0	0	0
Nakamurella_multipartita_DSM_44233_uid59221	1	0	0	0
Natrialba_magadii_ATCC_43099_uid46245	1	0	0	0
Natrinema_J7_uid171337	1	0	0	0
Natrinema_pellirubrum_DSM_15624_uid74437	1	0	0	0
Natronobacterium_gregoryi_SP2_uid74439	1	0	0	0
Natronococcus_occultus_SP4_uid184863	1	0	0	0
Natronomonas_pharaonis_DSM_2160_uid58435	1	0	0	0
Nitrobacter_hamburgensis_X14_uid58293	1	0	0	0
Nitrobacter_winogradskyi_Nb_255_uid58295	1	0	0	0
Nitrosococcus_halophilus_Nc4_uid46803	1	0	0	0
Nitrosococcus_oceani_ATCC_19707_uid58403	1	0	0	0
Nitrosococcus_watsonii_C_113_uid50331	1	0	0	0
Nitrosospira_multiformis_ATCC_25196_uid58361	1	0	0	0
Nocardia_brasiliensis_ATCC_700358_uid86913	1	0	0	0
Nocardia_farcinica_IFM_10152_uid58203	1	0	0	0
Nocardioides_JS614_uid58149	1	0	1	0
Novosphingobium_aromaticivorans_DSM_12444_uid5774	1	0	0	0
Novosphingobium_PP1Y_uid67383	1	0	0	0
Oceanimonas_GK1_uid81627	1	0	0	0
Oceanithermus_profundus_DSM_14977_uid60855	1	0	0	0
Oceanobacillus_iheyensis_HTE831_uid57867	1	0	0	0
Ochrobactrum_anthropi_ATCC_49188_uid58921	1	0	0	0
Octadecabacter_antarcticus_307_uid54701	1	0	0	0
Octadecabacter_arcticus_238_uid54699	1	0	0	0
Oligotropha_carboxidovorans_OM4_uid162135	1	0	0	0
Oligotropha_carboxidovorans_OM5_uid59155	1	0	0	0
Oligotropha_carboxidovorans_OM5_uid72795	1	0	0	0
Paenibacillus_JDR_2_uid59021	1	0	0	0
Paenibacillus_larvae_04_309_uid232355	1	0	0	0
Paenibacillus_mucilaginosus_3016_uid89377	1	0	0	0
Paenibacillus_mucilaginosus_KNP414_uid68311	1	0	0	0
Paenibacillus_Y412MC10_uid41127	1	0	0	0
Pandoraea_pnomenusa_3kgm_uid229878	1	0	0	0
Pandoraea_RB_44_uid231151	1	0	0	0
Parachlamydia_acanthamoebae_UV7_uid68335	1	0	0	0
Paracoccus_aminophilus_JCM_7686_uid214795	1	0	0	0
Paracoccus_denitrificans_PD1222_uid58187	1	0	0	0
Parvibaculum_lavamentivorans_DS_1_uid58739	1	0	0	0

Pedobacter_heparinus_DSM_2366_uid59111	1	0	0	0
Pedobacter_saltans_DSM_12145_uid61349	1	0	0	0
Pelagibacterium_halotolerans_B2_uid74393	1	0	0	0
Persicivirga_dokdonensis_DSW_6_uid186842	1	0	0	0
Phaeobacter_gallaeciensis_2_10_uid54715	1	0	0	0
Phaeobacter_gallaeciensis_DSM_17395_uid54717	1	0	0	0
Phaeobacter_gallaeciensis_DSM_26640_uid232357	1	0	0	0
Phenylobacterium_zucineum_HLK1_uid58959	1	0	0	0
Photobacterium_profundum_SS9_uid62923	1	0	0	0
Picrophilus_torridus_DSM_9790_uid58041	1	0	0	0
Pleurocapsa_PCC_7327_uid183006	1	0	0	0
Polaribacter_MED152_uid54207	1	0	0	0
Polaromonas_JS666_uid58207	1	0	0	0
Polaromonas_naphthalenivorans_CJ2_uid58273	1	0	0	0
Polymorphum_gilvum_SL003B_26A1_uid65447	1	0	0	0
Polynucleobacter_necessarius_asymbioticus_QLW_P1DM	1	0	0	0
Polynucleobacter_necessarius_STIR1_uid58967	1	0	0	0
Pseudoalteromonas_atlantica_T6c_uid58283	1	0	0	0
Pseudoalteromonas_haloplanktis_TAC125_uid58431	1	0	0	0
Pseudoalteromonas_SM9913_uid61247	1	0	0	0
Pseudogulbenkiania_NH8B_uid73423	1	0	0	0
Pseudomonas_aeruginosa_B136_33_uid196598	1	0	0	0
Pseudomonas_aeruginosa_DK2_uid168996	1	0	0	0
Pseudomonas_aeruginosa_LES431_uid232245	1	0	0	0
Pseudomonas_aeruginosa_LESB58_uid59275	1	0	0	0
Pseudomonas_aeruginosa_M18_uid162089	1	0	0	0
Pseudomonas_aeruginosa_MTB_uid231150	1	0	0	0
Pseudomonas_aeruginosa_NCGM2_S1_uid162173	1	0	0	0
Pseudomonas_aeruginosa_PA1R_uid228932	1	0	0	0
Pseudomonas_aeruginosa_PA1_uid228931	1	0	0	0
Pseudomonas_aeruginosa_PAO1_uid57945	1	0	0	0
Pseudomonas_aeruginosa_RP73_uid209328	1	0	0	0
Pseudomonas_aeruginosa_SCV20265_uid232358	1	0	0	0
Pseudomonas_aeruginosa_UCBPP_PA14_uid57977	1	0	0	0
Pseudomonas_brassicacearum_NFM421_uid66303	1	0	0	0
Pseudomonas_denitrificans_ATCC_13867_uid195459	1	0	0	0
Pseudomonas_entomophila_L48_uid58639	1	0	0	0
Pseudomonas_fluorescens_A506_uid165185	1	0	0	0
Pseudomonas_fluorescens_CHA0_uid203393	1	0	0	0
Pseudomonas_fluorescens_F113_uid87037	1	0	0	0
Pseudomonas_fluorescens_Pf0_1_uid57591	1	0	0	0
Pseudomonas_fluorescens_Pf_5_uid57937	1	0	0	0
Pseudomonas_fluorescens_SBW25_uid158693	1	0	0	0
Pseudomonas_fulva_12_X_uid67351	1	0	0	0
Pseudomonas_mendocina_ymp_uid58723	1	0	0	0
Pseudomonas_monteilii_SB3078_uid232252	1	0	0	0
Pseudomonas_monteilii_SB3101_uid232253	1	0	0	0
Pseudomonas_ND6_uid167583	1	0	0	0
Pseudomonas_poae_RE_1_1_14_uid188480	1	0	0	0
Pseudomonas_putida_BIRD_1_uid162055	1	0	0	0
Pseudomonas_putida_DOT_T1E_uid171260	1	0	0	0
Pseudomonas_putida_F1_uid58355	1	0	0	0
Pseudomonas_putida_GB_1_uid58735	1	0	0	0
Pseudomonas_putida_H8234_uid208673	1	0	0	0
Pseudomonas_putida_HB3267_uid184078	1	0	0	0
Pseudomonas_putida_KT2440_uid57843	1	0	0	0
Pseudomonas_putida_NBRC_14164_uid208670	1	0	0	0
Pseudomonas_putida_S16_uid68747	1	0	0	0
Pseudomonas_putida_UW4_uid182733	1	0	0	0
Pseudomonas_putida_W619_uid58651	1	0	0	0
Pseudomonas_resinovorans_NBRC_106553_uid208671	1	0	0	0
Pseudomonas_stutzeri_A1501_uid58641	1	0	0	0
Pseudomonas_stutzeri_ATCC_17588___LMG_11199_uid	1	0	0	0
Pseudomonas_stutzeri_CCUG_29243_uid168379	1	0	0	0
Pseudomonas_stutzeri_DSM_10701_uid170940	1	0	0	0
Pseudomonas_stutzeri_DSM_4166_uid162113	1	0	0	0
Pseudomonas_stutzeri_RCH2_uid184342	1	0	0	0
Pseudomonas_syringae_phaseolicola_1448A_uid58099	1	0	0	0
Pseudomonas_TKP_uid232248	1	0	0	0
Pseudomonas_VLB120_uid226717	1	0	0	0

Pseudonocardia_dioxanivorans_CB1190_uid65087	1	0	0	0
Pseudovibrio_FO_BEG1_uid82373	1	0	0	0
Pseudoxanthomonas_spadix_BD_a59_uid75113	1	0	0	0
Psychrobacter_arcticus_273_4_uid58021	1	0	0	0
Psychrobacter_cryohalolentis_K5_uid58373	1	0	0	0
Psychrobacter_G_uid210641	1	0	0	0
Psychrobacter_PRwf_1_uid58459	1	0	0	0
Psychroflexus_torquis_ATCC_700755_uid54205	1	0	0	0
Psychromonas_CNPT3_uid54249	1	0	0	0
Psychromonas_ingrahamii_37_uid58521	1	0	0	0
Pusillimonas_T7_7_uid66391	1	0	0	0
Pyrobaculum_1860_uid82379	1	0	0	0
Pyrobaculum_aerophilum_IM2_uid57727	1	0	0	0
Pyrobaculum_arsenaticum_DSM_13514_uid58409	1	0	0	0
Pyrobaculum_calidifontis_JCM_11548_uid58787	1	0	0	0
Pyrobaculum_islandicum_DSM_4184_uid58635	1	0	0	0
Pyrobaculum_neutrophilum_V24Sta_uid58421	1	0	0	0
Pyrobaculum_oguniense_TE7_uid84411	1	0	0	0
Ralstonia_eutropha_H16_uid62925	1	0	1	0
Ralstonia_eutropha_JMP134_uid58047	1	0	0	0
Ralstonia_pickettii_12D_uid58859	1	0	0	0
Ralstonia_pickettii_12J_uid58737	1	0	0	0
Ralstonia_solanacearum_CFBP2957_uid50545	1	0	0	0
Ralstonia_solanacearum_CMR15_uid227773	1	0	0	0
Ralstonia_solanacearum_FQY_4_f_uid194089	1	0	0	0
Ralstonia_solanacearum_GMI1000_uid57593	1	0	0	0
Ralstonia_solanacearum_Po82_uid162133	1	0	0	0
Ralstonia_solanacearum_PSI07_uid50539	1	0	0	0
Ramlibacter_tataouinensis_TTB310_uid68279	1	0	0	0
Renibacterium_salmoninarum_ATCC_33209_uid58899	1	0	0	0
Rhizobium_etli_bv__mimosae_Mim1_uid213896	1	0	0	0
Rhizobium_etli_CFN_42_uid58377	1	0	0	0
Rhizobium_etli_CIAT_652_uid59115	1	0	0	0
Rhizobium_IRBG74_uid222820	1	0	0	0
Rhizobium_leguminosarum_bv__trifolii_WSM1325_uid589	1	0	0	0
Rhizobium_leguminosarum_bv__trifolii_WSM2304_uid589	1	0	0	0
Rhizobium_leguminosarum_bv__viciae_3841_uid57955	1	0	0	0
Rhizobium_NGR234_uid59081	1	0	0	0
Rhizobium_tropici_CIAT_899_uid185179	1	0	0	0
Rhodobacter_sphaeroides_2_4_1_uid57653	1	0	0	0
Rhodobacter_sphaeroides_ATCC_17025_uid58451	1	0	0	0
Rhodobacter_sphaeroides_ATCC_17029_uid58449	1	0	0	0
Rhodobacter_sphaeroides_KD131_uid59277	1	0	0	0
Rhodococcus_equi_103S_uid60171	1	0	0	0
Rhodococcus_erythropolis_CCM2595_uid216088	1	0	0	0
Rhodococcus_erythropolis_PR4_uid59019	1	0	0	0
Rhodococcus_jostii_RHA1_uid58325	1	0	0	0
Rhodococcus_opacus_B4_uid13791	1	0	0	0
Rhodococcus_pyridinivorans_SB3094_uid232359	1	0	0	0
Rhodomicrobium_vannielii_ATCC_17100_uid43247	1	0	0	0
Rhodopseudomonas_palustris_BisA53_uid58445	1	0	0	0
Rhodopseudomonas_palustris_BisB18_uid58443	1	0	0	0
Rhodopseudomonas_palustris_BisB5_uid58441	1	0	0	0
Rhodopseudomonas_palustris_CGA009_uid62901	1	0	0	0
Rhodopseudomonas_palustris_DX_1_uid43327	1	0	0	0
Rhodopseudomonas_palustris_HaA2_uid58439	1	0	0	0
Rhodopseudomonas_palustris_TIE_1_uid58995	1	0	0	0
Rhodospirillum_centenum_SW_uid58805	1	0	0	0
Rhodospirillum_photometricum_uid159003	1	0	0	0
Rhodospirillum_rubrum_ATCC_11170_uid57655	1	0	0	0
Rhodospirillum_rubrum_F11_uid162149	1	0	0	0
Rhodothermus_marinus_DSM_4252_uid41729	1	0	0	0
Rhodothermus_marinus_SG0_5JP17_172_uid72767	1	0	0	0
Riemerella_anatipestifer_ATCC_11845___DSM_15868_ui	1	0	0	0
Riemerella_anatipestifer_ATCC_11845___DSM_15868_ui	1	0	0	0
Riemerella_anatipestifer_RA_CH_1_uid175469	1	0	0	0
Riemerella_anatipestifer_RA_CH_2_uid186548	1	0	0	0
Riemerella_anatipestifer_RA_GD_uid162013	1	0	0	0
Robiginitalea_biformata_HTCC2501_uid58285	1	0	0	0
Roseiflexus_castenholzii_DSM_13941_uid58287	1	0	0	0

Roseiflexus_RS_1_uid58523	1	0	0	0
Roseobacter_denitrificans_OCh_114_uid58597	1	0	0	0
Roseobacter_litoralis_Och_149_uid54719	1	0	0	0
Rubrivivax_gelatinosus_IL144_uid158163	1	0	0	0
Rubrobacter_xylanophilus_DSM_9941_uid58057	1	0	0	0
Ruegeria_pomeroyi_DSS_3_uid57863	1	0	0	0
Ruegeria_TM1040_uid58193	1	0	0	0
Ruminococcus_champanellensis_18P13_uid197169	1	0	0	0
Saccharomonospora_viridis_DSM_43017_uid59055	1	0	0	0
Saccharopolyspora_erythraea_NRRL_2338_uid62947	1	0	0	0
Salinarchaeum_laminariae_Harcht_Bsk1_uid207001	1	0	0	0
Salinibacter_ruber_M8_uid47323	1	0	0	0
Salinispora_arenicola_CNS_205_uid58659	1	0	0	0
Shewanella_amazonensis_SB2B_uid58257	1	0	0	0
Shewanella_ANA_3_uid58347	1	0	0	0
Shewanella_baltica_BA175_uid52601	1	0	0	0
Shewanella_baltica_OS117_uid162025	1	0	0	0
Shewanella_baltica_OS155_uid58259	1	0	0	0
Shewanella_baltica_OS185_uid58743	1	0	0	0
Shewanella_baltica_OS195_uid58261	1	0	0	0
Shewanella_baltica_OS223_uid58775	1	0	0	0
Shewanella_baltica_OS678_uid50553	1	0	0	0
Shewanella_denitrificans_OS217_uid58263	1	0	0	0
Shewanella_frigidimarina_NCIMB_400_uid58265	1	0	0	0
Shewanella_halifaxensis_HAW_EB4_uid59007	1	0	0	0
Shewanella_loihica_PV_4_uid58349	1	0	0	0
Shewanella_MR_4_uid58345	1	0	0	0
Shewanella_MR_7_uid58343	1	0	0	0
Shewanella_oneidensis_MR_1_uid57949	1	0	0	0
Shewanella_pealeana_ATCC_700345_uid58705	1	0	0	0
Shewanella_piezotolerans_WP3_uid58745	1	0	0	0
Shewanella_putrefaciens_200_uid161927	1	0	0	0
Shewanella_putrefaciens_CN_32_uid58267	1	0	0	0
Shewanella_sediminis_HAW_EB3_uid58835	1	0	0	0
Shewanella_violacea_DSS12_uid47085	1	0	0	0
Shewanella_W3_18_1_uid58341	1	0	0	0
Shewanella_woodyi_ATCC_51908_uid58721	1	0	0	0
Shigella_sonnei_Ss046_uid58217	1	0	0	0
Sideroxydans_lithotrophicus_ES_1_uid46801	1	0	0	0
Simiduia_agarivorans_SA1_uid177713	1	0	0	0
Singulisphaera_acidiphila_DSM_18658_uid81777	1	0	0	0
Sinorhizobium_fredii_HH103_uid86865	1	0	0	0
Sinorhizobium_fredii_USDA_257_uid168059	1	0	0	0
Sinorhizobium_medicae_WSM419_uid58549	1	0	0	0
Sinorhizobium_meliloti_1021_uid57603	1	0	0	0
Sinorhizobium_meliloti_2011_uid193772	1	0	0	0
Sinorhizobium_meliloti_AK83_uid52607	1	0	0	0
Sinorhizobium_meliloti_BL225C_uid52605	1	0	0	0
Sinorhizobium_meliloti_GR4_uid184823	1	0	0	0
Sinorhizobium_meliloti_Rm41_uid176372	1	0	0	0
Sinorhizobium_meliloti_SM11_uid159685	1	0	0	0
Solibacillus_silvestris_StLB046_uid168516	1	0	0	0
Solitalea_canadensis_DSM_3403_uid81783	1	0	0	0
Sphaerobacter_thermophilus_DSM_20745_uid41997	1	0	0	0
Sphingobacterium_21_uid64755	1	0	0	0
Sphingobium_chlorophenolicum_L_1_uid52597	1	0	0	0
Sphingobium_japonicum_UT26S_uid47077	1	0	0	0
Sphingomonas_MM_1_uid193771	1	0	0	0
Sphingomonas_wittichii_RW1_uid58691	1	0	0	0
Sphingopyxis_alaskensis_RB2256_uid58351	1	0	0	0
Staphylococcus_aureus_04_02981_uid161969	1	0	0	0
Staphylococcus_aureus_08BA02176_uid175257	1	0	0	0
Staphylococcus_aureus_11819_97_uid159981	1	0	0	0
Staphylococcus_aureus_55_2053_uid55909	1	0	0	0
Staphylococcus_aureus_6850_uid217772	1	0	0	0
Staphylococcus_aureus_71193_uid162141	1	0	0	0
Staphylococcus_aureus_Bmb9393_uid210640	1	0	0	0
Staphylococcus_aureus_CC45_uid209174	1	0	0	0
Staphylococcus_aureus_CN1_uid217769	1	0	0	0
Staphylococcus_aureus_COL_uid57797	1	0	0	0

Staphylococcus_aureus_ECT_R_2_uid159389	1	0	0	0
Staphylococcus_aureus_ED133_uid159689	1	0	0	0
Staphylococcus_aureus_ED98_uid41455	1	0	0	0
Staphylococcus_aureus_HO_5096_0412_uid162163	1	0	0	0
Staphylococcus_aureus_JH1_uid58457	1	0	0	0
Staphylococcus_aureus_JH9_uid58455	1	0	0	0
Staphylococcus_aureus_JKD6008_uid159855	1	0	0	0
Staphylococcus_aureus_JKD6159_uid159691	1	0	0	0
Staphylococcus_aureus_LGA251_uid159391	1	0	0	0
Staphylococcus_aureus_M013_uid88065	1	0	0	0
Staphylococcus_aureus_M1_uid197263	1	0	0	0
Staphylococcus_aureus_MRSA252_uid57839	1	0	0	0
Staphylococcus_aureus_MSHR1132_uid89393	1	0	0	0
Staphylococcus_aureus_MSSA476_uid57841	1	0	0	0
Staphylococcus_aureus_Mu3_uid58817	1	0	0	0
Staphylococcus_aureus_Mu50_uid57835	1	0	0	0
Staphylococcus_aureus_MW2_uid57903	1	0	0	0
Staphylococcus_aureus_N315_uid57837	1	0	0	0
Staphylococcus_aureus_NCTC_8325_uid57795	1	0	0	0
Staphylococcus_aureus_Newman_uid58839	1	0	0	0
Staphylococcus_aureus_SA40_uid221289	1	0	0	0
Staphylococcus_aureus_SA957_uid221288	1	0	0	0
Staphylococcus_aureus_ST228_10388_uid193754	1	0	0	0
Staphylococcus_aureus_ST228_10497_uid193755	1	0	0	0
Staphylococcus_aureus_ST228_15532_uid193756	1	0	0	0
Staphylococcus_aureus_ST228_16035_uid193757	1	0	0	0
Staphylococcus_aureus_ST228_18412_uid193760	1	0	0	0
Staphylococcus_aureus_ST398_uid159247	1	0	0	0
Staphylococcus_aureus_T0131_uid159861	1	0	0	0
Staphylococcus_aureus_TCH60_uid159859	1	0	0	0
Staphylococcus_aureus_TW20_uid159241	1	0	0	0
Staphylococcus_aureus_uid193758	1	0	0	0
Staphylococcus_aureus_uid193759	1	0	0	0
Staphylococcus_aureus_uid193761	1	0	0	0
Staphylococcus_aureus_USA300_TCH1516_uid58925	1	0	0	0
Staphylococcus_aureus_VC40_uid88071	1	0	0	0
Staphylococcus_aureus_Z172_uid225604	1	0	0	0
Staphylococcus_carnosus_TM300_uid59401	1	0	0	0
Staphylococcus_pseudintermedius_ED99_uid162109	1	0	0	0
Staphylococcus_pseudintermedius_HKU10_03_uid62125	1	0	0	0
Staphylococcus_saprophyticus_ATCC_15305_uid58411	1	0	0	0
Stenotrophomonas_maltophilia_D457_uid162199	1	0	0	0
Stenotrophomonas_maltophilia_JV3_uid72473	1	0	0	0
Stenotrophomonas_maltophilia_K279a_uid61647	1	0	0	0
Stenotrophomonas_maltophilia_R551_3_uid58657	1	0	0	0
Streptomyces_albus_J1074_uid196849	1	0	0	0
Streptomyces_avermitilis_MA_4680_uid57739	1	0	0	0
Streptomyces_bingchenggensis_BCW_1_uid82931	1	0	0	0
Streptomyces_cattleya_NRRL_8057___DSM_46488_uid1(	1	0	0	0
Streptomyces_cattleya_NRRL_8057_uid77117	1	0	0	0
Streptomyces_coelicolor_A3_2__uid57801	1	0	0	0
Streptomyces_collinus_Tu_365_uid214429	1	0	0	0
Streptomyces_davawensis_JCM_4913_uid193657	1	0	0	0
Streptomyces_flavogriseus_ATCC_33331_uid40839	1	0	0	0
Streptomyces_fulvissimus_DSM_40593_uid201038	1	0	0	0
Streptomyces_griseus_NBRC_13350_uid58983	1	0	0	0
Streptomyces_PAMC26508_uid197217	1	0	0	0
Streptomyces_rapamycinicus_NRRL_5491_uid227224	1	0	0	0
Streptomyces_scabiei_87_22_uid46531	1	0	0	0
Streptomyces_SirexAA_E_uid72627	1	0	0	0
Streptomyces_venezuelae_ATCC_10712_uid177080	1	0	0	0
Streptomyces_violaceusniger_Tu_4113_uid52609	1	0	0	0
Sulfobacillus_acidophilus_DSM_10332_uid88061	1	0	0	0
Sulfobacillus_acidophilus_TPY_uid68841	1	0	0	0
Sulfolobus_acidocaldarius_DSM_639_uid58379	1	0	0	0
Sulfolobus_acidocaldarius_N8_uid189027	1	0	0	0
Sulfolobus_acidocaldarius_Ron12_I_uid189028	1	0	0	0
Sulfolobus_acidocaldarius_SUSAZ_uid232254	1	0	0	0
Sulfolobus_islandicus_HVE10_4_uid162067	1	0	0	0
Sulfolobus_islandicus_LAL14_1_uid197216	1	0	0	0

Sulfolobus_islandicus_L_D_8_5_uid43679	1	0	0	0
Sulfolobus_islandicus_L_S_2_15_uid58871	1	0	0	0
Sulfolobus_islandicus_M_14_25_uid58849	1	0	0	0
Sulfolobus_islandicus_M_16_27_uid58851	1	0	0	0
Sulfolobus_islandicus_M_16_4_uid58841	1	0	0	0
Sulfolobus_islandicus_REY15A_uid162071	1	0	0	0
Sulfolobus_islandicus_Y_G_57_14_uid58923	1	0	0	0
Sulfolobus_islandicus_Y_N_15_51_uid58825	1	0	0	0
Sulfolobus_solfataricus_98_2_uid167998	1	0	0	0
Sulfolobus_solfataricus_P2_uid57721	1	0	0	0
Sulfolobus_tokodaii_7_uid57807	1	0	0	0
Syntrophobacter_fumaroxidans_MPOB_uid58177	1	0	0	0
Syntrophomonas_wolfei_Goettingen_uid58179	1	0	0	0
Syntrophothermus_lipocalidus_DSM_12680_uid49527	1	0	0	0
Thalassolituus_oleivorans_MIL_1_uid195604	1	0	0	0
Thauera_MZ1T_uid58987	1	0	0	0
Thermaerobacter_marianensis_DSM_12885_uid61727	1	0	0	0
Thermobifida_fusca_YX_uid57703	1	0	0	0
Thermomonospora_curvata_DSM_43183_uid41885	1	0	0	0
Thermoplasma_acidophilum_DSM_1728_uid61573	1	0	0	0
Thermoplasma_volcanium_GSS1_uid57751	1	0	0	0
Thermus_CCB_US3_UF1_uid81197	1	0	0	0
Thermus_oshimai_JL_2_uid178948	1	0	0	0
Thermus_scotoductus_SA_01_uid62273	1	0	0	0
Thermus_thermophilus_HB27_uid58033	1	0	0	0
Thermus_thermophilus_HB8_uid58223	1	0	0	0
Thermus_thermophilus_JL_18_uid162129	1	0	0	0
Thermus_thermophilus_SG0_5JP17_16_uid159537	1	0	0	0
Thioalkalivibrio_nitratireducens_DSM_14787_uid184011	1	0	0	0
Thiomonas_3As_uid178369	1	0	0	0
Thiomonas_intermedia_K12_uid48825	1	0	0	0
Tistrella_mobilis_KA081020_065_uid167486	1	0	0	0
Tsukamurella_paurometabola_DSM_20162_uid48829	1	0	0	0
Turneriella_parva_DSM_21527_uid168321	1	0	0	0
Variovorax_paradoxus_B4_uid218005	1	0	0	0
Variovorax_paradoxus_EPS_uid62107	1	0	0	0
Variovorax_paradoxus_S110_uid59437	1	0	0	0
Verminephrobacter_eiseniae_EF01_2_uid58675	1	0	0	0
Vibrio_alginolyticus_NBRC_15630___ATCC_17749_uid19	1	0	0	0
Vibrio_EJY3_uid83161	1	0	0	0
Vibrio_Ex25_uid41601	1	0	0	0
Vibrio_fischeri_ES114_uid58163	1	0	0	0
Vibrio_furnissii_NCTC_11218_uid82347	1	0	0	0
Vibrio_harveyi_ATCC_BAA_1116_uid58957	1	0	0	0
Vibrio_nigripulchritudo_SnF1_uid222819	1	0	0	0
Vibrio_parahaemolyticus_BB22OP_uid184822	1	0	0	0
Vibrio_parahaemolyticus_O1_K33_CDC_K4557_uid21297	1	0	0	0
Vibrio_parahaemolyticus_RIMD_2210633_uid57969	1	0	0	0
Vibrio_vulnificus_CMCP6_uid62909	1	0	0	0
Vibrio_vulnificus_MO6_24_O_uid62243	1	0	0	0
Vibrio_vulnificus_YJ016_uid58007	1	0	0	0
Vulcanisaeta_distributa_DSM_14429_uid52827	1	0	0	0
Vulcanisaeta_moutnovskia_768_28_uid63631	1	0	0	0
Waddlia_chondrophila_WSU_86_1044_uid49531	1	0	0	0
Weeksella_virosa_DSM_16922_uid63627	1	0	0	0
Xanthobacter_autotrophicus_Py2_uid58453	1	0	0	0
Xanthomonas_axonopodis_citri_306_uid57889	1	0	0	0
Xanthomonas_axonopodis_citrumelo_F1_uid73179	1	0	0	0
Xanthomonas_axonopodis_Xac29_1_uid193774	1	0	0	0
Xanthomonas_campestris_8004_uid57595	1	0	0	0
Xanthomonas_campestris_ATCC_33913_uid57887	1	0	0	0
Xanthomonas_campestris_uid61643	1	0	0	0
Xanthomonas_campestris_vesicatoria_85_10_uid58321	1	0	0	0
Xanthomonas_citri_Aw12879_uid194444	1	0	0	0
Xanthomonas_oryzae_KACC_10331_uid58155	1	0	0	0
Xanthomonas_oryzae_MAFF_311018_uid58547	1	0	0	0
Xanthomonas_oryzae_oryzicola_BLS256_uid54411	1	0	0	0
Xanthomonas_oryzae_PXO99A_uid59131	1	0	0	0
Xenorhabdus_nematophila_ATCC_19061_uid49133	1	0	0	0
Zobellia_galactanivorans_uid70621	1	0	0	0

Zunongwangia_profunda_SM_A87_uid48073	1	0	0	0

Table S2

The table contains a catalogue of all sequenced bacterial genomes which carry all genes for pyruvate pathway in context but lack the ETF subunit of butyryl-CoA dehydrogenase.
